# Prevalence and Implications of Shiga Toxin-Producing *E. coli* in Farm and Wild Ruminants

**DOI:** 10.3390/pathogens11111332

**Published:** 2022-11-11

**Authors:** Ritesh Ray, Pallavi Singh

**Affiliations:** Department of Biological Sciences, Northern Illinois University, Dekalb, IL 60115, USA

**Keywords:** Shiga toxin-producing *E. coli* (STEC), pathogen transmission, emerging serotypes, one-health

## Abstract

Shiga-toxin-producing *Escherichia coli* (STEC) is a food-borne pathogen that causes human gastrointestinal infections across the globe, leading to kidney failure or even death in severe cases. *E. coli* are commensal members of humans and animals’ (cattle, bison, and pigs) guts, however, may acquire Shiga-toxin-encoded phages. This acquisition or colonization by STEC may lead to dysbiosis in the intestinal microbial community of the host. Wildlife and livestock animals can be asymptomatically colonized by STEC, leading to pathogen shedding and transmission. Furthermore, there has been a steady uptick in new STEC variants representing various serotypes. These, along with hybrids of other pathogenic *E. coli* (UPEC and ExPEC), are of serious concern, especially when they possess enhanced antimicrobial resistance, biofilm formation, etc. Recent studies have reported these in the livestock and food industry with minimal focus on wildlife. Disturbed natural habitats and changing climates are increasingly creating wildlife reservoirs of these pathogens, leading to a rise in zoonotic infections. Therefore, this review comprehensively surveyed studies on STEC prevalence in livestock and wildlife hosts. We further present important microbial and environmental factors contributing to STEC spread as well as infections. Finally, we delve into potential strategies for limiting STEC shedding and transmission.

## 1. Introduction

**Figure 1:**
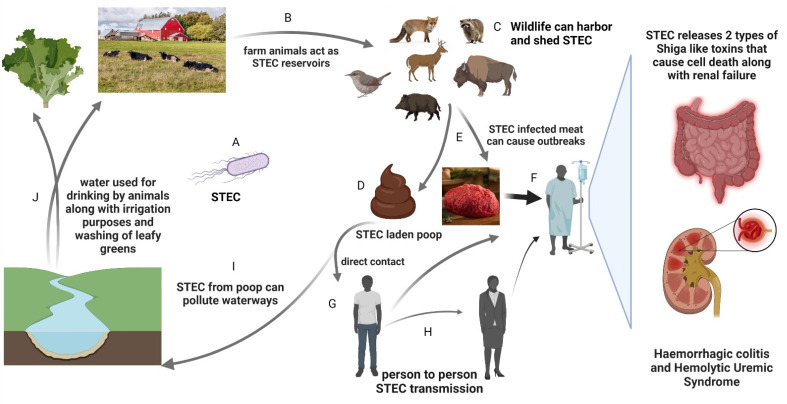
STEC transmission between livestock and wildlife reservoirs in the environment. (**A**) STEC (**B**) livestock are reservoirs of STEC and can shed the bacteria through contact with small mammals, bord and animal vectors; (**C**) wildlife can come in contact with vectors, harbor and spread STEC through (**D**) shedding via feces and (**E**) during meat processing. (**F**) Humans can also get infected by consuming STEC-infected meat and other animal products or (**G**) through direct contact with STEC-laden feces. All of these can lead to disease outbreaks of hemorrhagic colitis and hemolytic uremic syndrome in humans. (**H**) STEC can also spread from one human to another through contact. (**I**) STEC-laden feces can be washed into waterways that are (**J**) routinely utilized by other animals and for agricultural practices.

Shiga toxin-producing *Escherichia coli* (STEC) is a pathotype of *Escherichia coli* (*E. coli*) capable of causing serious ailments in humans and is estimated to cause 3890 fatal cases annually worldwide [1]. Although *E. coli* is a member of the natural gut flora of humans and animals, some pathotypes can cause ailments, including diarrhea, bloody diarrhea or hemorrhagic colitis (HC), kidney failure, or hemolytic uremic syndrome (HUS), and may be fatal in severe cases [2,3]. In addition, thrombotic thrombocytopenic purpura, a rare blood disease that occurs due to circulating Shiga toxin (Stx) leading to small blood clots, can also result from STEC infection in humans [4,5]. Overall, STEC causes 2,801,000 acute infections worldwide each year, with a greater proportion of instances (N = 809,000) in children below the age of 4, leading to $280 million in damage to the US healthcare industry [1,3]. Newborns and toddlers are especially at risk from STEC compared to healthy adults [6]. Even though the most common route of STEC infection is the consumption of undercooked meat and its products [7,8], it can be transferred from the mother’s gastrointestinal tract (GIT) to the child, as they are primary caregivers [9]. Additionally, contact with animals colonized with STEC has been reported to cause approximately one tenth of infections [10]. This bacterial pathogen asymptomatically colonizes the GIT of ruminants—such as cows, deer, and sheep, to name a few—and can be transmitted via zoonosis (Figure 1) [11,12]. Fecal–oral, food-borne, environmental, and person-to-person are all reported possible routes of STEC transmission (Figure 1) [8,13]. STEC asymptomatically colonizes adult ruminants, but may cause diarrhea in calves at a very low dosage [14,15]. STEC in ruminants can be passed to humans through beef during its processing and transport [16]. STEC is also responsible for huge economic loss in the meat processing industry, leading to two recalls in 2021 amounting to a loss of 300,096 pounds of meat [17]. There were also similar multiple contamination events in 2022 that led to multiple recalls of ground beef products [16,18,19]. Similarly, in post-weaned piglets, STEC-carrying Stx2e can cause edema disease (ED), a neurological disease resulting in high swine mortality [20,21]. ED affects fast-growing pigs, which leads to significant financial losses in the swine industry [20,21]. 

Due to dwindling natural areas, there is increased interaction between wild animals, humans, and domestic animals. This gives rise to the potential for transmission through either direct contact or fecal contamination of agriculture or game meat [22]. STEC can colonize both domestic and wild animals (e.g., deer, elk, sheep, boar, buffalo, goats, and fox) and shed from the animal during defecation [12,23,24]. These animals that shed bacteria through feces are known as shedders and can shed in the range of 10 to 10^9^ CFU/g (colony-forming units per gram) [25]. The animals that shed more than 10^4^ CFU/g are categorized as super-shedders and are responsible for transmitting to the entire herd through the fecal–oral route [25,26,27]. These super-shedders may also be responsible for transmission events across herds and animal species by contaminating water sources [27,28]. 

STEC can also evolve to colonize a new host or develop better transmission and environmental survival strategies [29,30]. Furthermore, due to increased interaction between reservoirs of STEC and humans, outbreaks and sporadic cases are becoming more common. Therefore, there is an increase in the incidence of zoonotic diseases, which requires a “One Health” approach. The One Health approach aims to sustainably regulate and optimize the health of humans, animals, and the ecosystem they reside in by addressing the demand for clean and nutritious food, water, and air [31]. This strategy mobilizes numerous sectors, disciplines, and communities at various societal levels to form collaborations to promote well-being and address risks to human health and ecosystems [31]. STEC is an important pathogen that must be addressed under One Health research due to its presence in various niches in the ecosystem. The goal of this review is to comprehensively compile bacterial, host, and environmental factors important for the transmission and infection of STEC. We further provide recommendations for checking STEC colonization in ruminants to help mitigate super-shedding events.

## 2. Virulence Factors Associated with STEC

STEC utilizes various virulence strategies to survive and proliferate in reservoir hosts as well as humans. In the section below, we summarize bacterial virulence factors that are important for STEC pathogenesis. STEC carries major virulence and antimicrobial resistance genes on mobile genetic elements that are important for pathogenesis. These virulence factors, therefore, can be shared through horizontal gene transfer (HGT), leading to the emergence of newer virulence strategies and pathotypes (Figure 1) [32]. Therefore, STEC outbreak detection requires systematic surveillance of these virulence genes.

### 2.1. Virulence Factors Important for Adherence and Colonization

The primary virulence factor for colonization, intimin, is encoded by the *eae* gene. This gene is essential for close bacterial adherence to epithelial cells, which results in “attaching and effacing” (A/E) histopathological lesions [33]. The *eae* gene is located in the locus of enterocyte effacement (LEE), a large pathogenicity island, which also encodes for the Type III secretion system (T3SS) responsible for A/E lesions on intestinal cells [34,35]. The T3SS secretes bacterial LEE and non-LEE effectors into the host cell, which leads to hemolysis, cytotoxicity, iron sequestration, destruction of microvilli, inhibition of apoptosis, and interference with inflammatory signaling pathways (inhibition of phagocytosis (EspF, EspH, and EspJ) [36]. A subset of STECs that are LEE-negative have loci of adhesion and autoaggregation (LAA), another pathogenicity island, that promotes colonization and infections in humans [37,38]. These, LEE-negative STECs therefore are able to utilize other forms of adhesion, including the aggregative adherence fimbriae (AAFs), regulated by *aggR* [39,40]; autoagglutinating adhesin (*saa*) [41], a plasmid-encoded enterohemolysin *ehxa* [42]; and the autotransporter gene *sab*, which is involved in biofilm formation [43]. Colonization of STEC in livestock animals and its shedding is dependent on various factors, such as host genetics, age, diet intake, and diversity and richness of the intestinal microbiome [44,45]. STEC shedding in colonized beef calves was negatively correlated to animal maturity, and shedding was more likely to occur during the first six months [44]. Once ingested, STEC manages to survive the low-pH and oxygen environment of the rumen and passes into the recto-anal junction (RAJ) for colonization [46]. Two adhesin genes have been described in STEC extensively, namely *efa1* and *eibG.* These genes aid in colonization at the RAJ [47]. The protein Efa1 was found to be essential for STEC to adhere to bovine epithelial cells, leading to colonization and subsequent shedding [48,49]. Another adhesin EibG (*E. coli*), immunoglobulin-binding protein G, aids in bacterial adhesion to epithelial cells [50]. Flagellin (*fliC*) and Lon protease (*lon*) help in bacterial movement and survival while in bacteriostatic conditions due to antibacterial agents [51]. A novel Type V secreted protein called extracellular serine protease (*espP*) has also been found to positively influence the adherence of STEC to the bovine colon mucosa [52].

### 2.2. Shiga Toxins Important for Pathogenesis

The main virulence factor of STEC is the Shiga toxin (*stx*) gene encoded on temperate bacteriophages (Stx phages) that causes HUS and HC [34,53,54,55]. Stx is an AB5 toxin that affects the host’s microvascular endothelial cell surfaces of the kidney, intestine, and brain in humans. Globotriaosylceramide (Gb3) receptors that have an affinity for the pentameric B subunit are expressed on the surface of these organs [56]. Upon attachment, the A subunit is released into the host cell cytoplasm, where it binds to the 28S RNA of the 60S ribosomal subunit. This leads to the inhibition of protein synthesis and apoptosis [2,53,56,57,58]. Based on the type of *stx* gene encoded by the phages, they are characterized either as Stx1 or Stx2 phages. STEC exhibit differences in virulence based on the stx phage type present. For instance, Stx2-carrying strains were reported to be more virulent and more frequently linked to HUS than strains carrying only Stx1 or both Stx1 and Stx2 phages [34]. Furthermore, Stx1 phages (7.6%) have been reported to be outnumbered by Stx2-phagepositive (68.4%) overall in samples from human and animal wastewater, feces, river water, soil, sludge, and food [59,60]. Stx2 phages can be induced by stressors, such as antibiotics [55,61], whereas Stx1 phages exhibit lower induction rates, which may explain the fewer free Stx1 phages in the environment compared to Stx2 [55]. This implies there would be a higher chance of acquiring a *stx2* than a *stx1* phage. Multiple variants of Stx1 (stx1a, stx1c, and stx1d) and Stx2 (stx2a, stx2b, stx2c, stx2d, stx2e, stx2f, and stx2g) have also been reported [62]. These variants have been linked to differences in clinical outcomes and toxicity. For instance, Stx2a was found to be more virulent than Stx1 with LD_50_ (in mice) at 6.5 ng compared to >1000 ng of Stx1 [63]. Stx variants have also been similarly correlated with disease severity. For instance, some *stx2* subtypes—such as *stx2a*, *stx2c,* and *stx2d*—are frequently linked to a higher risk of developing HUS, whereas others—such as *stx2e*, *stx2b*, *stx2f,* and *stx2g*—have been linked to milder diseases [63,64,65]. 

### 2.3. Other Toxigenic Virulence Factors

In STEC, toxin production is also carried out by *cdt-V* (cytolethal distending toxin -V), *astA* (EAST-1 toxin), *subAB* (subtilin toxin), and *estA* (ETEC thermo-stable toxin) [66,67]. Lipopolysaccharide (LPS), an endotoxin, activates the complement system through the lectin pathway [68]. This leads to the production of the chemotactic anaphylatoxins C3a and C5a, which can destroy the kidney and other internal organs [69]. This cascade further leads to the activation of TNF-alpha, cytokines, and chemokines, triggering an acute inflammatory response and host tissue damage [69,70].

### 2.4. Biofilms

STECs use ruminants as reservoirs, and their presence in livestock and food processing factories has been well documented [71]. It has been proposed that STECs’ ability to form biofilms on various surfaces is responsible for their transmission and persistence in food processing facilities [72]. Biofilm, a key mechanism for bacterial survival, can be formed on a wide range of solid surfaces by secreting various surface proteins and extracellular-matrix components (EPS) [72]. An important protein, curli, forms proteinaceous extracellular fibers that help in cell–cell interactions to support cell aggregation, biofilm formation, and host colonization [73,74]. This was evident from the importance of curli fimbriae demonstrated in STEC O157:H7 adhesion to bovine recto-anal epithelial cells [75], leafy greens, alfalfa sprouts, and stainless steel [76,77]. Biofilm formation, dependent on the EPS components, is highly regulated by at least six proteins encoded by the csgBA and csgDEFG operons and various two-component systems (OmpR/EnvZ, CpxA/R, and Rcs) [74,78,79,80,81]. However, genetic comparison between strong- and weak-biofilm-forming STEC differed through a small number (between 0 and 13) of single nucleotide polymorphisms (SNP), indicating high genetic similarity between strains colonizing livestock [27].

In livestock, biofilm has been implicated in STEC dispersal [28]; STEC isolated from cattle have high capabilities of forming biofilm that can be sloughed off from the host’s intestines, leading to super-shedding events [25,27]. These super-shedding events can lead to the contamination of food, which results due to contact with livestock fecal matter [82]. Therefore, STEC has been isolated from contaminated vegetables, such as romaine lettuce, lettuce, unpasteurized apple cider or juice, melon, spinach, radish sprouts, and alfalfa sprouts [83,84]. Certain STEC strains possess biological mechanisms unique to their interactions with lettuce leaves, including genes that could play a key role in biofilm formation and regulation [85]. 

In food processing facilities, biofilms are found on the floors, walls, pipelines, and drains [86,87]. STEC can form biofilms on a wide range of materials, including stainless steel, aluminum, nylon, Teflon, rubber, plastic, glass, and polyurethane, which are frequently used in food processing equipment [86]. As a result, animal-protein-related products, such as ground beef, roast beef, ground bison, and salami, along with animal fats and related products, such as raw milk, cheese, ice cream, and yogurt, have all been recognized as carriers of STEC [88,89]. Furthermore, STEC is protected against sanitizing treatments due to EPS generation [72]. When antimicrobial treatment is applied to the biofilm, persister cells form which can tolerate high levels of antibacterial compounds [90]. These persister cells continue to form biofilms after the treatment has ceased through close aggregation; they are transmitted between reservoirs and infect humans [90]. Therefore, continuous molecular surveillance of STEC from various sources and fomites is imperative. This will help in checking the proliferation and persistence of STEC to ultimately assist in reducing transmission and infection rates.

## 3. Major Seropathotypes of STEC and Its Constituents

**Figure 2:**
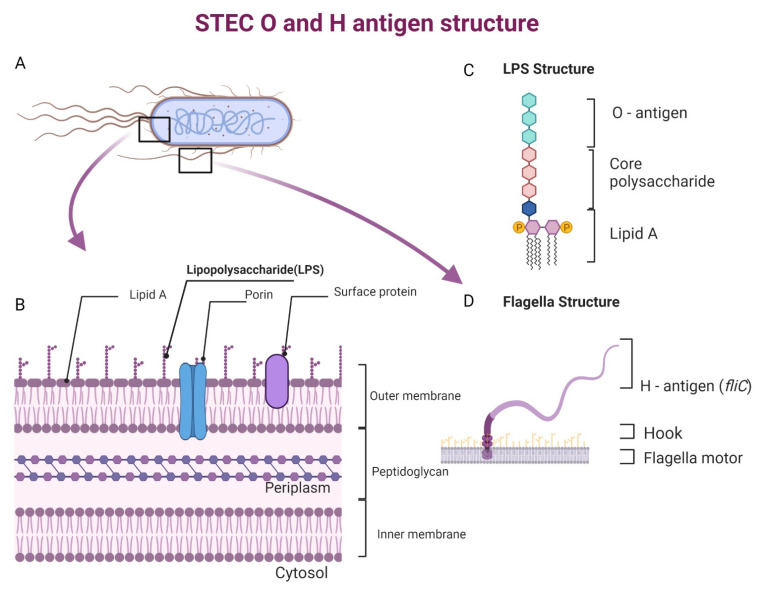
Structure of antigens present on Shiga toxin *E. coli* (**A**) depiction of a STEC bacterium; (**B**) gram-negative bacterial cell wall structure as present in STEC with the presence of various polysaccharide, including lipopolysaccharide (LPS) on its surface; (**C**) Structure of LPS consists of lipid A, core polysaccharide and O-antigen; (**D**) Strudcute of flagella s as found in gram-negative bacteria such as STEC is made up of a motor, hook, and the flagellar H-antigen.

To date, more than 470 serogroups of *E. coli* have been found to carry genes that encode for either Stx1, 2, or both [91,92]. STEC strains of the same serotype may still carry a variety of virulence genes on mobile genetic components that can be lost or transferred [93]. Serotypes of STEC are defined by characterizing the somatic antigen (O) comprised of O-side chain sugar molecules (Figure 2C) and the flagellar antigen (H) (Figure 2D) [94]. The variations in both O and H antigens determine the immunological specificity and aid in pathogenesis [94,95]. For instance, peritrichous flagella on STEC cell surface have been implicated in early biofilm formation by binding to bovine host proteins, such as mucins, mucus, and extracellular matrix proteins, promoting colonization [96,97]. STEC serotypes reported to be involved in disease outbreaks mainly include O157 and the non-O157 serotypes called the “Big six” (O26, O45, O103, O111, O121, and O145) [98]. Even though STEC O157 has been historically related to outbreaks, recent trends have reported a higher number of outbreaks originating from non-O157 serotypes [34]. Non-O157 serotypes have developed as major enteric pathogens in nations such as Japan, Argentina, Chile, Germany, Australia, the United States, Canada, and Ireland within the last 15 years [34,99,100,101]. In this section, we report prevalent serotypes and their sources and implications. 

### 3.1. Serotypes of STEC prevalent in Livestock

Livestock animals have been colonized with the majority of STEC serotypes isolated to date. Consumption of beef is implicated as the second-most frequent cause of foodborne outbreaks in the US [102]. Livestock raised for beef have been reported to harbor the big six STEC serotypes (Table 1) [103,104]. STEC contamination has been found in numerous samples of uncooked retail meat from animals raised for consumption in China, with O128, O176, and O91 found to be most prevalent [105]. In total, 373 livestock-associated STEC serotypes have been found; 65 have been detected in HUS patients, while 62 cause other human illnesses [103]. Various other serotypes—such as O2, O5, O8, O22, O91, O171, O15, O113, and O174—have been isolated from retail raw meat, livestock feces, and farms, which also possess antibiotic resistance (Table 1) [28,34,105,106]. During a 2019 study EU, O13, O55, and O91 were the three most prevalent serogroups in fresh bovine meat [107]. The serotypes that carry the *stx* gene and LEE encoding genes have been responsible for fatal cases of hemolytic uremic syndrome in humans (Table 1) [34]. It is important to surveil serotypes that can cause HUS in humans and have become prevalent in food sources in multiple countries, such as O91 and O113 isolated in livestock [108,109]. Contact with livestock or consuming beef or mutton has been associated with the highest rate of HUS (37%) while infected by non-O157 STEC [110]. The feed used for raising poultry was found to be spiked with various STEC serotypes, such as O26, O103, O111, O121, O145, O157:H7, and several untypeable serotypes [111], showing potential STEC outbreak potential from poultry farms. Livestock such as goats carry serogroups such as O93 with a newer variant of *stx—stx2k*—which has increased in prevalence over time [112]. Although results are not clear on the severity of the *stx2k* toxin on humans, it could still cause disease outbreaks in the future due to homology with other *stx* genes [113,114]. New emergent STEC serotypes, such as O145 from livestock, have become prevalent in some populations such as the UK and have shown a higher frequencies of admittance due to infection than O157 along with a higher HUS rate than O157 [115].

### 3.2. Serotypes of STEC in Wild Animals

Wild animals can play a major role as reservoirs and super-shedders of STEC in nature [121,132]. STEC O157 has been isolated from wild deer species in multiple geographical locations [121,124,125]. Due to increased wildlife interactions with agricultural land, products, and waste, there is potential for wildlife to become major STEC reservoirs. It should be noted that different wildlife species also harbor lesser-known or studied STEC serotypes [121,133]. Along with the top seven STEC serotypes, other non-O157 serotypes, such as O2, O5, O8, O22, O113, O91, and O174, are some of the serotypes of STEC strains isolated from wildlife animals (Table 1) [121]. The serogroup O8 was found to harbor a variant of *stx*—*stxf*—first isolated from wild pigeons [134] as well as *eae*, which was able to cause HUS in the Netherlands [135]. Human-neonatal-disease-causing serotypes, such as O146, were found in deer meat in the EU in 2015 [9,136]. Wild boar meat, along with some unspecified meat samples, were found positive for non-O157 STEC in the same year in the EU [136]. More European studies found cultivatable non-O157 STEC serotypes, such as O27, O146, and O178, in 17% of wild animals, including antibiotic-resistant O103 from red foxes [137]. These serogroups also showed a closed evolutionary linkage between clinical isolates of STEC, thus having a high potential to cause an outbreak [137]. Often, serotypes isolated from wild animals have varied genetic makeups compared to their counterparts in livestock, which leads to lower detection through conventional methods [64,65]. Several other serotypes of STEC beyond the scope of this review occur in wildlife but with varied prevalence.

### 3.3. Serotypes of STEC in Environment

Although STEC is pervasive in domestic and wild animals, open spaces, and agricultural settings, certain serotypes have been reported in specific environments (Table 1). Various environmental factors are responsible for the spread of STEC, and once shed by an animal, it can survive for a long time in the environment [138]. In a study, STEC was detectable in four beef cow barns from three different fairgrounds for a duration of 10 months or more after the fair had ended [139]. Rainfall has the potential to wash agricultural waste and effluents into water sources laden with STEC, which has the potential to infect different hosts in numerous ways (Figure 1) [10]. Environmental STEC transmission can also happen through contact with recreational water [140] or water used for aquaculture [122]. In fresh fish, shellfish, and their ready-to-eat products sold in retail markets, investigations have found both O157 and non-O157 STEC (Table 1) [122,141]. STEC spreads in an agricultural setting when contaminated water is used for agricultural irrigation. It can lead to contamination of agricultural produce, such as spinach, lettuce, cilantro, and alfalfa sprouts, which leads to losses through callbacks of these products (Figure 1) [118]. STEC contamination in agricultural produce and meat is becoming more common due to the increased incidence of pathogenic serotypes, such as O113, in the environment [142]. The serotypes mentioned in Table 1 have also been isolated from wildlife and environmental samples, making their study a higher priority in the One Health approach. 

## 4. Emerging STEC Serotypes 

**Figure 3:**
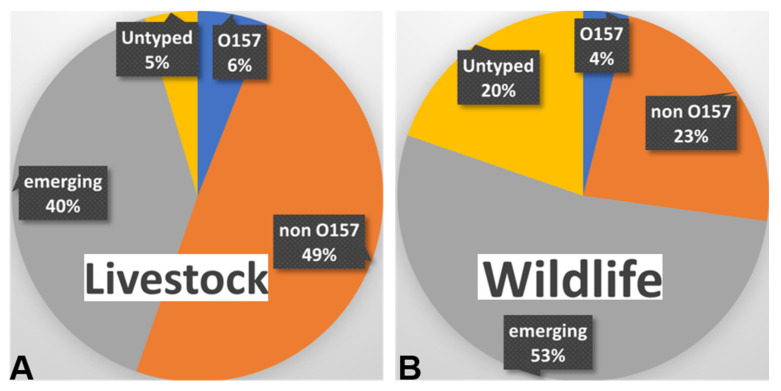
Pie charts depicting combined global STEC prevalence in (**A**) livestock vs. (**B**) wildlife animals and their surrounding based on serotype. Data are compiled from both groups and include the prevalence of O157, non-O157 (O26, O45, O103, O111, O121, O145), emerging serotypes (O113, O2, O5, O8, O91, O168, etc.), and untyped STEC serotypes. Data are based on References [29,66,67,99,100,101,130,132,133,143,145,146,147,148,149,150,151,152,155,156,157,158,159,160,161,162,163,164].

Due to the dynamic transmission of STEC between various reservoirs and genetic variations due to constant selective pressure, there are several emerging STEC serotypes, including the ones that were previously reported in a different host and environment. These emerging serotypes often carry multi-drug-resistant genes [143,144]. From studies conducted from 2016–2022, STEC non-O157—along with other emergent serotypes, such as O2, O5, O8, O15, O22, O91, O113, O171, and O174—has increased in prevalence in both wildlife and livestock studies (Figure 3). STEC serotypes belonging to non-O157 (big six and other prevalent serotypes listed in Table 1) and untyped serotypes have become more prevalent over the last 5 years [29,66,67,130,132,145,146,147,148,149,150,151,152]. In an Argentine study, O174:H21, O185:H7, O8:H19, O178:H19, and O130:H11 represented 42.5% of the isolates from beef abattoirs [100]. The serotypes O2:H6, O5:HNM, O21:H21, O26:[H11], O36:H14, O110:H45, O128:H2, O146:[H21], O146:[H28], O174:[H8], ONT:HNT [95,120], O100, O97, O91, O149, O92, O102, and O34 are non-O157 serotypes prevalent in swine [147]. Stx phages have been found to have high genetic similarity (81–100%) while infecting a wide range of *E. coli* pathotypes and serotypes (O2, O111, and O168) isolated from various sources (i.e., cattle, humans, and food) [114,142,153,154]. There are reports of the emergence of hybrid strains carrying both STEC and enterotoxigenic *E. coli* (ETEC) virulence factors [113]. These hybrids, along with *stx2k*, have heat-labile toxins (*elt*), making their pathogenic potential severe [112,113]. In this section, we expand on various causes for emergence of new and hypervirulent serotypes.

### 4.1. Wildlife as Food Choices (Game Meat)

The rising numbers of wild grazing mammals can hurt the environment and agriculture through overgrazing [165]. A useful way to make use of wild animals as a natural resource is through the marketing of game meat as a food source. Consumers that prioritize taste, nutritional value, and low fat content in the evaluation of products make up a consumer group who may choose to eat game meat more frequently [166]. Wildlife hunted for game meat, such as deer, elk, moose, and wildebeest, have been shown to harbor and spread STEC. It becomes important that we keep hunters and game meat enthusiasts safe from sporadic outbreaks from STEC. These interactions between disease-carrying wildlife and recreational hunters will increase in the future. There are currently no guidelines for the handling and consumption of free-range game meat in North America [167]. As consumption of game meat increases in North America, this trend may increase the risk of STEC and other foodborne illnesses for recreational hunters and others who consume game meat [168]. 

### 4.2. Interspecies Transmission

STEC has been found and reported in newer host animals, although ruminants are typically the main source of STEC pathotypes [34]. Bison and water buffalo also have been implicated in harboring STEC [169,170]. Spillover hosts are secondary species that act as a host when exposed to the STEC by close contact with ruminants or feeding supplies contaminated with ruminant excrement [171]. They are generally transitory hosts of STEC and harbor the organism through continuous reinfections from a super-shedder organism. Livestock animals, such as sheep, goats, horses, and swine, are recognized as spillover hosts [171,172,173]. STEC can contaminate water sources near livestock operations which in certain instances are simultaneously utilized for aquaculture, leading to STEC transmission in fisheries [141,174]. These findings support the hypotheses that fish and shellfish may serve as STEC reservoirs or spillover hosts. Domestic and wild birds have also been implicated in being carriers of STEC [175,176]. In a study conducted on a wide range of wild birds, STEC and its virulence genes were found [176]. The birds access to large areas and bird droppings can help spread STEC to newer host niches.

### 4.3. Rise in Antibiotic-Resistant Strains

An increase in STEC antibiotic resistance is another major issue that we are facing currently. STEC resistant to various broad-spectrum antibiotics has been isolated from livestock, wildlife sources, and the environment [30,67,177,178]. The use of antibiotic growth promoters (AGPs) has further enhanced this problem of antibiotic resistance in STEC. Since their initial proposal in 1946 for use as growth promoters in livestock [179], AGPs have been administered to livestock at subtherapeutic concentrations to bolster their growth rate and feed conversion efficiency [180,181,182]. As the livestock industry expanded, so did the usage of AGPs for increased yield demands of meat [180]. Multi-drug resistant STEC O157 and non-O157 were isolated from cattle feces where AGPs were used as feed additives [183,184,185]. Antibiotics such as ciprofloxacin (CIP), trimethoprim-sulfamethoxazole, and fluoroquinolones can generate an SOS response in vitro in STEC strains by targeting DNA synthesis [186,187]. This causes induction of the Stx phage and enhanced Stx toxin production [187]. Due to this, there is a high possibility of the evolution of non-pathogenic *E. coli* (i.e., non-STEC) to STEC in the intestines of cattle following antibiotic treatment. Exposure to manure contaminated with leftover antibiotics is also possible and may lead to the transduction of Stx-encoding phages and the acquisition of antibiotic resistance [186]. There is a strong likelihood that these antibiotic-resistant strains will enter the environment and eventually spread the resistance genes to nonpathogenic environmental bacteria [182,183]. Further extended spectrum beta lactamase-producing STEC have been observed at the highest concentrations in the soil near livestock, followed by drinking water for the livestock and effluents from the farm [143]. Colistin (Polymyxin E), a cationic polypeptide antibiotic that has historically been allowed for use in animals used for food due to the low resistance rates, has slowly become less effective in infected animals and humans due to increased bacterial resistance [23,188]. Colistin resistance in various bacteria has been discovered to be carried by a conjugative plasmid and is easily transmissible between diverse bacterial populations [188]. Further studies demonstrated that bacterial isolates detected in pigs have transferred this plasmid-based resistance to other strains of bacteria [23,188,189,190].

Various clinical treatment approaches have been previously compiled for STEC infections [191]. To treat STEC infections in humans, antibiotics such as rifaximin, fosfomycin, azithromycin, and meropenem are administered only in the early stages of infection [192,193]. Antibiotics that target DNA synthesis can cause Stx phage induction inside the host, leading to higher toxin production. Therefore, these antibiotics are not recommended for treating STEC infections. Because STEC is subjected to the same selective pressures in the host and environment as any other bacterium, antibiotic administration may enhance antibiotic resistance among these foodborne pathogens [194]. For instance, resistance to azithromycin may limit our ability to use therapeutic medicines to inhibit STEC [195]. Alternatives to antibiotic treatments, such as phage therapy, might be an option in the future [196,197]. 

## 5. Control of STEC Transmission

As noted in previous sections, STEC can be dynamically transmitted between various sources and ultimately to humans. There are several checkpoints along this route that can be controlled to prevent transmission and infection. For example, contact with either livestock or their feces should be regarded as a potential source of STEC transmission, as they have been linked to disease outbreaks [198]. In this section, we provide various measures that can be taken to control STEC transmission between sources and, more importantly, between livestock and wildlife. These measures are also important for limiting the proliferation of emerging STEC serotypes and multi-drug-resistant strains.

### 5.1. Transmission Control in Livestock

Temperature, dietary changes, age, herd size, and access to wildlife have all been correlated with STEC transmission and shedding in livestock [199]. Beef cattle have been associated with higher shedding compared to dairy cattle [199]. Lactation was identified as a stress factor correlated with higher STEC prevalence and shedding, especially in dairy cattle [200]. Herd management incorporating proper hygiene and sanitizing chemical treatment of animal effluents with enhanced precautions given to lactating cows may lower the probability of STEC shedding and transmission. Diet is important for promoting livestock health via mass gain and modulating pathogen colonization. The addition of feed additives with proven antimicrobial properties, such as seaweed extract and Tasco-14, has lowered the incidence of STEC O157 in cattle feces and hide [201]. Therefore, dietary supplements that focus not only on mass gain but also on reduced pathogen colonization and shedding should be administered in farm-raised animals. To manage STEC shedding in livestock, especially cattle, different forms of vaccines have also been employed, resulting in a change in fecal shedding [202]. Vaccines that targeted bacterial survival factors, such as the T3SS (*p* value = 0.0009), and Siderophore receptor, and porin protein (SRP) (*p* value < 0.01), showed a significant reduction in fecal shedding in cattle [203]. Insects have also been deemed vectors to pathogenic *E. coli* which can lead to higher transmission of the bacterium in livestock herds [204,205]. Herd management practices to control the proliferation of insect vectors, such as flies, can potentially reduce STEC transmission within and between herds.

### 5.2. Control of Transmission to Wildlife 

Due to increased interaction between wildlife and agricultural settings, wild animals have become reservoirs and/or spillover hosts. A recent important example is SARS-CoV-2 (severe acute respiratory syndrome-coronavirus 2), which has been detected in white-tailed deer, highlighting the importance of spillover transmission [170]. Similarly, hunters that hunt wildlife for game meat have been implicated in STEC transmission [171,172]. Farmers who may also be hunters are an especially important group that must be trained through outreach regarding the importance of transmission [152,175,206]. Likewise, butcher shops/abattoirs where game meat is processed have been implicated in infection outbreaks multiple times [173]. Stx phages from super-shedders can be transduced into *E. coli* carried by insects to make hybrid variants that may be able to infect different hosts [207,208]. Culling high densities of insect and filth flies would be ideal to slow the down large-scale transmission of STA. Lower prevalence of filth flies can be achieved via fumigation or topical application of essential oils derived from thyme and catnip in the living quarters of the livestock [209,210]. Dogs and other vertebrate scavengers, such as civets, were able to drive down the interaction between wildlife and cattle by consuming dead animal carcasses, reducing resource availability for harmful bacteria and their insect vectors to breed and initiate interspecies transfer [199]. Processing wildlife meat, such as venison or elk or bison meat, should require continuous surveillance for STEC prevalence and be included under the surveillance program through the CDC. 

### 5.3. Transmission Control in Food Processing Facilities

The ability of both O157 and non-O157 STEC stereotypes to form biofilms on stainless steel makes them ideal adulterants in the food factory setting [45]. Rigorous cleaning regimens and the use of antibiotic agents, such as chlorine dioxide (ClO2) and sodium hypochlorite (NaOCl), can help in limiting biofilm formation in processing facilities. Some STEC strains have been found to persist on surfaces after the application of antibiotic treatments due to biofilm formation [174]. By utilizing the curli extracellular matrix, STEC can outcompete other bacteria in mixed-species biofilms [44]. STEC has an advantage in colonizing processing plants and infecting large quantities of meat processed through these multibacterial biofilms. STEC had higher resistance to sanitization even with low EPS expression when forming mixed biofilms with a companion EPS-producing Salmonella strain [175]. This is because bacterial EPS components can protect their companion bacterial strains regardless of species in mixed biofilms while simultaneously enhancing the EPS-producing strains’ resistance to sanitizers [175]. Thus, STEC is routinely associated with multiple other bacterial species to form complex biofilms in processing plants [175,176]. Food acids, such as sodium acid sulfate and nonionic surfactants called polysorbates, have been utilized to slow down STEC transmission by slowing the biofilm formation of various STEC serotypes [211,212]. Natural products including plant secondary metabolites—such as flavonoids, terpenes, and essential oils—have been employed to inhibit biofilm formation in STEC [213]. Since AGPs lead to an increase in antibiotic resistance, using phytochemicals in livestock settings will diminish antibiotic resistance [214]. As feed additives, phytochemicals can replace antibiotic growth promoters (AGP), which are typically used in livestock production [214,215]. There are different kinds of phytochemicals—such as flavonoids, terpenes, and essential oils—that have been employed to inhibit STEC biofilm [216,217,218,219,220]. The use of flavonoids, such as coumarin and ginkgolic acid, have also shown suppression of the curli gene and autoinducers of STEC, leading to reduced biofilm formation and shedding [218,219]. Essential oils and terpenes can also inhibit the biofilm formation of STEC and keep vectors (insects) at bay while boosting gut health and feed conversion [220,221,222].

## 6. Role of the Intestinal Microbiome in Colonization and Infections

To infect humans, STEC must reach the GI tract and outcompete the indigenous microbial population for colonization. In this section, we look at the role of human host-related factors such as diet and microbiome that contribute to the development of STEC infection. Various methods of preventing STEC infection and outbreaks have also been highlighted. We also discuss human host factors that may make them more susceptible to STEC colonization and severe infections. 

### 6.1. Intestinal Microbial Communities of Reservoir Animals

The cattle hindgut microbiome contains two major prevalent microbial groups, *Firmicutes* and *Bacteriodetes*, followed by other microbial communities, such as *Spirochaetes* and *Proteobacteria* [223]. Prevotella and Treponema were more prevalent in STEC non-shedders [224], while Ruminococcus and Selenomonas were found to be more prevalent in STEC super-shedders when microbiota were sampled from GIT tissues and content at the slaughterhouse [224]. STEC can also transiently influence the host’s microbial community for ease in colonization [225]. This is supported by the significant difference (*p* value < 0.05) in the bacterial community structure of both fecal and RAJ between shedders and non-shedders [225,226]. It was also reported that cattle animals with higher STEC prevalence have higher alpha diversity of intestinal microorganisms [223,225]. Diet was found to be a key determinant of intestinal microbiota in the animal and important for STEC shedding [227,228,229]. For example, a high-fiber and forage diet was linked to higher STEC shedding in comparison to diets high in steam-flaked maize or grains [45,200]. Grain-based diets resulted in a higher abundance of Proteobacteria and a lower abundance of Bacteriodetes, whereas forage-dominant diets were found to have a high abundance of Firmicutes, Ruminococcaceae, and *Paludibacter*, critical for degrading forage [230]. Sudden dietary changes from forage- to grain-based or hay-based to grain-based were correlated with higher STEC shedding in cattle [231]. STEC shedding was also shown to increase significantly (*p* value < 0.05) when distillers grains, a high-energy and protein source, exceed 40% of the cattle diet [232]. 

### 6.2. Perturbed Intestinal Microbiome Leads to STEC Infection in Humans

The gastrointestinal (GI) tract of an adult human has about 500–1000 species of microorganisms, amounting to 10^14^ bacteria [233]. The typical human gut microbiota is dominated by the phyla *Firmicutes*, *Bacteroidetes*, *Actinobacteria*, and *Proteobacteria* [233,234]. Cross-regulation between the host and the gut microbiota keeps the GI tract healthy and prevents the proliferation of potentially harmful bacteria by preserving a homeostatic balance of microorganisms [233]. If this typical human gut microbiome changes in its composition, it is termed dysbiosis [235]. This perturbed/dysbiotic microbiota can give rise to enteric infections due to selection pressures in the microbiome, such as inflammatory bowel disease (IBD), allergies, diabetes, obesity, and multiple sclerosis [235,236,237].

During enteric infections—including those caused by STEC—due to selection pressures, Proteobacteria, especially *Escherichia,* outcompeted other microbes and become a major community in the intestines [236]. Furthermore, STEC and other pathogenic microorganisms can take advantage of dysbiosis of the intestinal microbiome and cause severe infections [238]. During an infection, the greatest shift in microbial population was seen in *Bacteroidetes*, *Firmicutes*, and *Proteobacteria* [236]. Dysbiosis is followed by inflammation of the intestinal tract, which leads to the production of host-derived nitrates [239]. These nitrates boost the growth of *E. coli* in intestines, causing even more dysbiosis among host microbiota, which may lead to higher rates of STEC infection [239]. 

It has also been observed that children, having lower intestinal microbiome diversity, have been associated with more STEC infections compared to adults [240]. Thus, a mature or diverse microbiome can confer protection against STEC colonization and infection. Furthermore, the healthy control group had a higher prevalence of growth-promoting microbial community members, such as *Bifidobacteriales* and *Clostridiales*, in comparison to the STEC-positive group [241]. These members are also capable of inhibiting pathogen colonization. In addition to this direct inhibition, the indigenous intestinal microbiome can indirectly affect factors such as pH, and secondary metabolites also compete for nutrients with the STEC in the host intestine [242]. In particular, microorganisms of *the Bacteroides* genus have been credited with not only stopping inflammatory reactions due to toxins produced by STEC but also protecting the host from death in a gnotobiotic mouse model [243]. Significant changes in dietary intake reflect on the host intestinal flora. A healthy microbiota, characterized by a high diversity of microbes as well as a high abundance of phyla such as *Bacteroidetes* and *Firmicutes,* defends against invading STEC pathogens [236,237]. A healthy, diverse diet supplemented with probiotics rich in bacterial communities such as *Bacteriodetes* and *Firmicutes* may help slow down STEC infection and colonization in the human intestines. 

A high-fiber diet has been linked to increased butyrate production in the intestine [244]. Increased production of butyrate, although found to promote colonic health, can lead to higher Gb3 expression in intestinal linings. This enhanced Stx toxin binding and uptake within the intestine during STEC infection leads to higher lethality during the course of the disease [244]. Furthermore, STEC secretes a AB5 toxin known as Subtilase cytotoxin (SubAB), which has shown a high affinity towards glycans, such as α2 –3-linked N-glycolylneuraminic acid (Neu5Gc) [245,246]. Therefore, humans with higher amounts of these glycans suffer higher toxin damage [246] due to their diets being able to integrate it into their intestinal epithelium and kidney vasculature [247]. Dairy products and red meat are the two main sources of Neu5Gc and are also the most frequent sources of STEC contamination. In other murine studies, it has been demonstrated that a diet high in fiber leads to the proliferation of symbiotic microbes such as *Akkermansia,* which have been reported to reduce inflammation caused by STEC infection or toxins and regulate intestinal health [227,248]. Therefore, dietary decisions can affect susceptibility to the toxin as well as likelihood of contracting the pathogen [249].

## 7. Conclusions

The study of STEC and its serotypes is important as there has been a steady uptick in sporadic infections of novel origin. The review has highlighted the importance of the increase in the number of serotypes beyond the most prevalent big six serotypes (Figure 3). Newer serotypes are emerging that harbor the *stx* gene and have not been typed yet [118,250]. There has also been a high incidence of multi-drug resistance in STEC isolates recently [29,151,152]. Outbreaks originating from these multi-drug-resistant untyped serotypes can cause severe healthcare disasters, as it would be difficult to pinpoint the outbreaks’ sources. The increase in emerging and untyped STEC serotypes is possibly linked to searching for newer habitats due to change in climatic conditions and shifts in availability of resources by both humans and animals. Human and animal encroachment into new habitats can lead to increased interaction with wildlife carrying STEC and related hybrids. Zoonotic pathogens, such as STEC O157, can exploit these conditions to their favor and cause repeated infections through various sources over different geographic locations [7,251,252,253]. Similarly, the non-O157 serotypes have also been increasingly linked to human infections [254]. 

Environmental transmission of STEC is amplified by ruminant reservoirs that can act as supper-shedders of STEC as well as Stx phages [255]. Untreated effluents and feedlots, which are generally full of feces, can evolve as a hot zone for STEC infection and transmission. Stx phages can withstand a variety of harsh conditions, including UV light, high temperatures, and sanitizing agents such as chlorine, suggesting that Stx phages can survive multiple inactivating events [256]. In addition, lateral gene transfer can occur in biofilms in a temperate bacteriophage-mediated process [257]. Transduction of *stx2* in biofilms was found to be highly temperature-dependent [257]. As climate change warms the environment, transduction and transmission processes are sped up, producing newer STEC variants with variable genetic makeups. To address the increased burden of emerging STEC serotypes, specific virulence genes characterization can be implemented for the detection of STEC at low levels in various reservoirs of STEC and food sources. The increased surveillance of STEC through molecular detection of virulence genes will proactively save human lives and prevent loss of livestock due to sporadic infections. 

As foodborne zoonotic pathogens, STEC are becoming more prevalent in the environment and more resistant to antibiotics, thereby leading to higher infection rates. Newer variants of STEC are being isolated from newer sources, warranting the need for frequent and robust molecular screening techniques. The One Health approach is needed to track STEC infections and must be updated regularly for newer virulence markers to keep up with rapidly evolving and emerging bacteria for molecular analysis. As the causes of contamination from STEC and its biofilm-associated pathogens skyrocket, we would need to address several different problems with stopping sporadic infections in the environment, such as reducing livestock shedding through established options such as vaccines while investing in more novel options. Historically, the livestock sector has been implicated in the high shedding of STEC, but recent trends show wildlife harboring and shedding STEC. If this transmission between two different habitats is not controlled, STEC can further evolve to become a more powerful pathogen harboring new combinations of virulence factors. To tackle the problem of rising variations of STEC virulence and infections, more research into events that lead to interspecies bacterial transmission—especially between wildlife and livestock—is warranted. 

## Figures and Tables

**Table 1:** STEC categorized by serotypes reported from various sources.

Serotypes	Livestock	Wildlife	Agriculture	Food Industry	Continental Prevalence	Source
O157	+	+	+	+	EU, NA, SA, AS, AF	[34,116,117,118,119,120]
Non-O157 (Big-6)						
O26	+	+	+	+	EU, NA, SA, AS, AF	[34,116,117,118,119,120,121,122]
O45	+	+	+	+	EU, NA, SA, AS, AF	[116,117,123,124]
O103	+	+	+	+	EU, NA, SA, AS, AF	[116,117,119,120,124]
O111	+	+		+	EU, NA, SA, AS, AF	[116,117,119,121,122,125]
O121	+	+		+	EU, NA, SA, AS, AF	[116,120,123]
O145	+	+		+	EU, NA, SA, AS, AF	[34,116,117,119,120,124]
Other Non-O157						
O113	+	+	+		EU, NA, SA, AS, AF	[34,109,118,126]
O2	+	+	+	+	EU, NA, SA, AS, AF	[118,120,123,127]
O5	+	+			EU, AF, AA, NA, SA	[120,128]
O8	+	+	+	+	NA, EU, SA, AS	[34,118,120,121,123,127,129]
O22	+	+			EU, NA, AS, SA	[67,100,121,130]
O91	+	+		+	EU, NA, SA, AS, AF	[34,119,121]
O171	+			+	EU, SA	[34,131]
O15	+			+	NA, SA	[4,123]
O174	+	+			EU, SA	[120,121,127]

STEC serotypes that were reported from various sources with more than 90% prevalence are included. Livestock includes cattle, sheep, goats, poultry, etc. Wildlife includes red deer, wild boar, otters, mouflons, feral birds, and red foxes. NA = North America, SA = South America, AF = Africa, AS = Asia, EU = Europe.

## Data Availability

Not applicable.

## References

[1] Majowicz S.E., Scallan E., Jones-Bitton A., Sargeant J.M., Stapleton J., Angulo F.J., Yeung D.H., Kirk M.D. (2014). Global incidence of human Shiga toxin–producing *Escherichia coli* infections and deaths: A systematic review and knowledge synthesis. Foodborne Pathog. Dis..

[2] Kaper J.B., Nataro J.P., Mobley H.L. (2004). Pathogenic *Escherichia coli*. Nat. Rev. Microbiol..

[3] Hoffmann S., Batz M.B., Morris Jr J.G. (2012). Annual cost of illness and quality-adjusted life year losses in the United States due to 14 foodborne pathogens. J. Food Prot..

[4] Xia X., Meng J., McDermott P.F., Ayers S., Blickenstaff K., Tran T.-T., Abbott J., Zheng J., Zhao S. (2010). Presence and characterization of Shiga toxin-producing *Escherichia coli* and other potentially diarrheagenic *E. coli* strains in retail meats. Appl. Environ. Microbiol..

[5] Tarr P.I. (2009). Shiga toxin-associated hemolytic uremic syndrome and thrombotic thrombocytopenic purpura: Distinct mechanisms of pathogenesis. Kidney Int..

[6] Crump J.A., Sulka A.C., Langer A.J., Schaben C., Crielly A.S., Gage R., Baysinger M., Moll M., Withers G., Toney D.M. (2002). An outbreak of *Escherichia coli* O157: H7 infections among visitors to a dairy farm. N. Engl. J. Med..

[7] Rivas M., Sosa-Estani S., Rangel J., Caletti M.G., Vallés P., Roldán C.D., Balbi L., de Mollar M.C.M., Amoedo D., Miliwebsky E. (2008). Risk factors for sporadic Shiga toxin–producing *Escherichia coli* infections in children, Argentina. Emerg. Infect. Dis..

[8] Kintz E., Brainard J., Hooper L., Hunter P. (2017). Transmission pathways for sporadic Shiga-toxin producing *E. coli* infections: A systematic review and meta-analysis. Int. J. Hyg. Environ. Health.

[9] Stritt A., Tschumi S., Kottanattu L., Bucher B.S., Steinmann M., von Steiger N., Stephan R., Hächler H., Simonetti G.D. (2013). Neonatal hemolytic uremic syndrome after mother-to-child transmission of a low-pathogenic stx 2b harboring Shiga toxin–producing *Escherichia coli*. Clin. Infect. Dis..

[10] Heiman K.E., Mody R.K., Johnson S.D., Griffin P.M., Gould L.H. (2015). *Escherichia coli* O157 outbreaks in the United States, 2003–2012. Emerg. Infect. Dis..

[11] Karmali M.A., Mascarenhas M., Shen S., Ziebell K., Johnson S., Reid-Smith R., Isaac-Renton J., Clark C., Rahn K., Kaper J.B. (2003). Association of Genomic O Island 122 of *Escherichia coli* EDL 933 with Verocytotoxin-Producing *Escherichia coli* Seropathotypes That Are Linked to Epidemic and/or Serious Disease. J. Clin. Microbiol..

[12] Espinosa L., Gray A., Duffy G., Fanning S., McMahon B.J. (2018). A scoping review on the prevalence of Shiga-toxigenic *Escherichia coli* in wild animal species. Zoonoses Public Health.

[13] Marder E.P., Cieslak P.R., Cronquist A.B., Dunn J., Lathrop S., Rabatsky-Ehr T., Ryan P., Smith K., Tobin-D’Angelo M., Vugia D.J. (2017). Incidence and trends of infections with pathogens transmitted commonly through food and the effect of increasing use of culture-independent diagnostic tests on surveillance—Foodborne Diseases Active Surveillance Network, 10 US sites, 2013–2016. Morb. Mortal. Wkly. Rep..

[14] Besser T., Richards B., Rice D., Hancock D. (2001). *Escherichia coli* O157 [ratio] H7 infection of calves: Infectious dose and direct contact transmission. Epidemiol. Infect..

[15] Mercado E., Gioffré A., Rodríguez S., Cataldi A., Irino K., Elizondo A., Cipolla A.L., Romano M.I., Malena R., Mendez M. (2004). Non-O157 Shiga toxin-producing *Escherichia coli* isolated from diarrhoeic calves in Argentina. J. Vet. Med. Ser. B.

[16] US Department of Agriculture, USDA FSIS Issues Public Health Alert for Ground Beef Products Due to Possible *E. coli* O157:H7 Contamina-tion. https://www.fsis.usda.gov/recalls-alerts/fsis-issues-public-health-alert-ground-beef-products-due-possible-e--coli-o157h7.

[17] US Department of Agriculture, USDA Summary of Recall Cases in Calendar Year 2021. https://www.fsis.usda.gov/food-safety/recalls-public-health-alerts/annual-recall-summaries/summary-recall-cases-calendar-8.

[18] US Department of Agriculture, USDA Lakeside Refrigerated Services Recalls Ground Beef Products Due to Possible *E. coli* O103 Con-tamination. https://www.fsis.usda.gov/recalls-alerts/lakeside-refrigerated-services-recalls-ground-beef-products-due-possible-e--coli.

[19] US Department of Agriculture, USDA FSIS Issues Public Health Alert for Specific Ground Beef in HelloFresh Meal Kits Due to Possible *E. coli* O157:H7 Contamination. https://www.fsis.usda.gov/recalls-alerts/fsis-issues-public-health-alert-specific-ground-beef-hellofresh-meal-kits-due.

[20] Cornick N.A., Matise I., Samuel J.E., Bosworth B.T., Moon H.W. (2000). Shiga toxin-producing *Escherichia coli* infection: Temporal and quantitative relationships among colonization, toxin production, and systemic disease. J. Infect. Dis..

[21] Tabaran F., Tabaran A. (2019). Edema disease of swine: A review of the pathogenesis. Porc. Res..

[22] Jones B.A., Grace D., Kock R., Alonso S., Rushton J., Said M.Y., McKeever D., Mutua F., Young J., McDermott J. (2013). Zoonosis emergence linked to agricultural intensification and environmental change. Proc. Natl. Acad. Sci. USA.

[23] Shafiq M., Huang J., Ur Rahman S., Shah J.M., Chen L., Gao Y., Wang M., Wang L. (2019). High incidence of multidrug-resistant *Escherichia coli* coharboring mcr-1 and bla (CTX-M-15) recovered from pigs. Infect. Drug Resist.

[24] Bertelloni F., Cagnoli G., Biagini F., Poli A., Bibbiani C., Ebani V.V. (2022). Virulence Genes of Pathogenic *Escherichia coli* in Wild Red Foxes (*Vulpes vulpes*). Animals.

[25] Munns K.D., Selinger L.B., Stanford K., Guan L., Callaway T.R., McAllister T.A. (2015). Perspectives on super-shedding of *Escherichia coli* O157: H7 by cattle. Foodborne Pathog. Dis..

[26] Matthews L., Low J., Gally D., Pearce M., Mellor D., Heesterbeek J., Chase-Topping M., Naylor S., Shaw D., Reid S. (2006). Heterogeneous shedding of *Escherichia coli* O157 in cattle and its implications for control. Proc. Natl. Acad. Sci. USA.

[27] Blankenship H.M., Carbonell S., Mosci R.E., McWilliams K., Pietrzen K., Benko S., Gatesy T., Grooms D., Manning S.D. (2020). Genetic and phenotypic factors associated with persistent shedding of Shiga toxin-producing *Escherichia coli* by beef cattle. Appl. Environ. Microbiol..

[28] Bumunang E.W., McAllister T.A., Zaheer R., Ortega Polo R., Stanford K., King R., Niu Y.D., Ateba C.N. (2019). Characterization of non-O157 *Escherichia coli* from cattle faecal samples in the North-West Province of South Africa. Microorganisms.

[29] Murphy R., Palm M., Mustonen V., Warringer J., Farewell A., Parts L., Moradigaravand D. (2021). Genomic epidemiology and evolution of *Escherichia coli* in wild animals in Mexico. Msphere.

[30] Yar A., Choudary M.A., Rehman A., Hussain A., Elahi A., ur Rehman F., Waqar A.B., Alshammari A., Alharbi M., Nisar M.A. (2022). Genetic Diversity and Virulence Profiling of Multi-Drug Resistant *Escherichia coli* of Human, Animal, and Environmental Origins. Antibiotics.

[31] One Health High-Level Expert P., Adisasmito W.B., Almuhairi S., Behravesh C.B., Bilivogui P., Bukachi S.A., Casas N., Cediel Becerra N., Charron D.F., Chaudhary A. (2022). One Health: A new definition for a sustainable and healthy future. PLoS Pathog..

[32] Glaize A., Gutierrez-Rodriguez E., Hanning I., Díaz-Sánchez S., Gunter C., van Vliet A.H.M., Watson W., Thakur S. (2020). Transmission of antimicrobial resistant non-O157 *Escherichia coli* at the interface of animal-fresh produce in sustainable farming environments. Int. J. Food Microbiol..

[33] Paton J.C., Paton A.W. (1998). Pathogenesis and diagnosis of Shiga toxin-producing *Escherichia coli* infections. Clin. Microbiol. Rev..

[34] Etcheverria A.I., Padola N.L. (2013). Shiga toxin-producing *Escherichia coli*: Factors involved in virulence and cattle colonization. Virulence.

[35] Mahanti A., Samanta I., Bandyopadhyay S., Joardar S. (2015). Molecular characterization and antibiotic susceptibility pattern of caprine Shiga toxin producing-*Escherichia coli* (STEC) isolates from India. Iran. J. Vet. Res..

[36] Wong A.R.C., Pearson J.S., Bright M.D., Munera D., Robinson K.S., Lee S.F., Frankel G., Hartland E.L. (2011). Enteropathogenic and enterohaemorrhagic *Escherichia coli*: Even more subversive elements. Mol. Microbiol..

[37] Montero D.A., Velasco J., Del Canto F., Puente J.L., Padola N.L., Rasko D.A., Farfán M., Salazar J.C., Vidal R. (2017). Locus of adhesion and autoaggregation (LAA), a pathogenicity island present in emerging Shiga toxin–producing *Escherichia coli* strains. Sci. Rep..

[38] Colello R., Vélez M.V., González J., Montero D.A., Bustamante A.V., Del Canto F., Etcheverría A.I., Vidal R., Padola N.L. (2018). First report of the distribution of Locus of Adhesion and Autoaggregation (LAA) pathogenicity island in LEE-negative Shiga toxin-producing *Escherichia coli* isolates from Argentina. Microb. Pathog..

[39] Nagy A., Xu Y., Bauchan G.R., Shelton D.R., Nou X. (2016). Aggregative adherence fimbriae I (AAF/I) mediate colonization of fresh produce and abiotic surface by Shiga toxigenic enteroaggregative *Escherichia coli* O104:H4. Int. J. Food Microbiol..

[40] Boisen N., Melton-Celsa A.R., Scheutz F., O’Brien A.D., Nataro J.P. (2015). Shiga toxin 2a and enteroaggregative *Escherichia coli*–a deadly combination. Gut Microbes.

[41] Paton A.W., Srimanote P., Woodrow M.C., Paton J.C. (2001). Characterization of Saa, a novel autoagglutinating adhesin produced by locus of enterocyte effacement-negative Shiga-toxigenic *Escherichia coli* strains that are virulent for humans. Infect. Immun..

[42] Lorenz S.C., Son I., Maounounen-Laasri A., Lin A., Fischer M., Kase J.A. (2013). Prevalence of hemolysin genes and comparison of ehxA subtype patterns in Shiga toxin-producing *Escherichia coli* (STEC) and non-STEC strains from clinical, food, and animal sources. Appl. Environ. Microbiol..

[43] Herold S., Paton J.C., Paton A.W. (2009). Sab, a Novel Autotransporter of Locus of Enterocyte Effacement-Negative Shiga-Toxigenic *Escherichia coli* O113:H21, Contributes to Adherence and Biofilm Formation. Infect. Immun..

[44] Mir R.A., Weppelmann T.A., Elzo M., Ahn S., Driver J.D., Jeong K.C. (2016). Colonization of Beef Cattle by Shiga Toxin-Producing *Escherichia coli* during the First Year of Life: A Cohort Study. PLoS ONE.

[45] Vasco K., Nohomovich B., Singh P., Venegas-Vargas C., Mosci R.E., Rust S., Bartlett P., Norby B., Grooms D., Zhang L. (2021). Characterizing the cattle gut microbiome in farms with a high and low prevalence of shiga toxin producing *Escherichia coli*. Microorganisms.

[46] Sapountzis P., Segura A., Desvaux M., Forano E. (2020). An Overview of the Elusive Passenger in the Gastrointestinal Tract of Cattle: The Shiga Toxin Producing *Escherichia coli*. Microorganisms.

[47] Sandt C.H., Hill C.W. (2000). Four different genes responsible for nonimmune immunoglobulin-binding activities within a single strain of *Escherichia coli*. Infect. Immun..

[48] Stevens M.P., van Diemen P.M., Frankel G., Phillips A.D., Wallis T.S. (2002). Efa1 influences colonization of the bovine intestine by Shiga toxin-producing *Escherichia coli* serotypes O5 and O111. Infect. Immun..

[49] Nicholls L., Grant T.H., Robins-Browne R.M. (2000). Identification of a novel genetic locus that is required for in vitro adhesion of a clinical isolate of enterohaemorrhagic *Escherichia coli* to epithelial cells. Mol. Microbiol..

[50] Merkel V., Ohder B., Bielaszewska M., Zhang W., Fruth A., Menge C., Borrmann E., Middendorf B., Müthing J., Karch H. (2010). Distribution and phylogeny of immunoglobulin-binding protein G in Shiga toxin-producing *Escherichia coli* and its association with adherence phenotypes. Infect. Immun..

[51] Stromberg Z.R., Masonbrink R.E., Mellata M. (2020). Transcriptomic Analysis of Shiga Toxin-Producing *Escherichia coli* during Initial Contact with Cattle Colonic Explants. Microorganisms.

[52] Dziva F., Mahajan A., Cameron P., Currie C., McKendrick I.J., Wallis T.S., Smith D.G.E., Stevens M.P. (2007). EspP, a Type V-secreted serine protease of enterohaemorrhagic *Escherichia coli* O157:H7, influences intestinal colonization of calves and adherence to bovine primary intestinal epithelial cells. FEMS Microbiol. Lett..

[53] Boerlin P., McEwen S.A., Boerlin-Petzold F., Wilson J.B., Johnson R.P., Gyles C.L. (1999). Associations between virulence factors of Shiga toxin-producing *Escherichia coli* and disease in humans. J. Clin. Microbiol..

[54] Schmidt H. (2001). Shiga-toxin-converting bacteriophages. Res. Microbiol..

[55] Allison H.E. (2007). Stx-phages: Drivers and mediators of the evolution of STEC and STEC-like pathogens. Future Med..

[56] Ivarsson M.E., Leroux J.C., Castagner B. (2012). Targeting bacterial toxins. Angew. Chem. Int. Ed..

[57] Karch H., Tarr P.I., Bielaszewska M. (2005). Enterohaemorrhagic *Escherichia coli* in human medicine. Int. J. Med. Microbiol..

[58] Sandvig K. (2001). Shiga toxins. Toxicon.

[59] Grau-Leal F., Quirós P., Martínez-Castillo A., Muniesa M. (2015). Free S higa toxin 1-encoding bacteriophages are less prevalent than S higa toxin 2 phages in extraintestinal environments. Environ. Microbiol..

[60] Crespo-Medina M., Greaves I., Hunter P.R., Minnigh H., Ramírez-Toro G. (2020). Detection of Shiga toxin-encoding genes in small community water supplies. J. Water Health.

[61] Tozzoli R., Grande L., Michelacci V., Ranieri P., Maugliani A., Caprioli A., Morabito S. (2014). Shiga toxin-converting phages and the emergence of new pathogenic *Escherichia coli*: A world in motion. Front. Cell. Infect. Microbiol..

[62] Scheutz F., Teel L.D., Beutin L., Piérard D., Buvens G., Karch H., Mellmann A., Caprioli A., Tozzoli R., Morabito S. (2012). Multicenter evaluation of a sequence-based protocol for subtyping Shiga toxins and standardizing Stx nomenclature. J. Clin. Microbiol..

[63] Fuller C.A., Pellino C.A., Flagler M.J., Strasser J.E., Weiss A.A. (2011). Shiga toxin subtypes display dramatic differences in potency. Infect. Immun..

[64] Krüger A., Lucchesi P.M.A. (2015). Shiga toxins and stx phages: Highly diverse entities. Microbiology.

[65] Friesema I., Van Der Zwaluw K., Schuurman T., Kooistra-Smid M., Franz E., Van Duynhoven Y., Van Pelt W. (2014). Emergence of *Escherichia coli* encoding Shiga toxin 2f in human Shiga toxin-producing *E. coli* (STEC) infections in the Netherlands, January 2008 to December 2011. Eurosurveillance.

[66] Valle C.-D., Mariana D., la Garza-García D., Jorge A., Díaz-Aparicio E., Valdivia-Flores A.G., Cisneros-Guzmán L.F., Rosario C., Manjarrez-Hernández Á.H., Navarro A. (2016). Characterization of *Escherichia coli* strains from red deer (*Cervus elaphus*) faeces in a Mexican protected natural area. Eur. J. Wildl. Res..

[67] Alonso C.A., Mora A., Díaz D., Blanco M., González-Barrio D., Ruiz-Fons F., Simón C., Blanco J., Torres C. (2017). Occurrence and characterization of stx and/or eae-positive *Escherichia coli* isolated from wildlife, including a typical EPEC strain from a wild boar. Vet. Microbiol..

[68] Baines A.C., Brodsky R.A. (2017). Complementopathies. Blood Rev.

[69] Orth D., Khan A.B., Naim A., Grif K., Brockmeyer J., Karch H., Joannidis M., Clark S.J., Day A.J., Fidanzi S. (2009). Shiga toxin activates complement and binds factor H: Evidence for an active role of complement in hemolytic uremic syndrome. J. Immunol..

[70] Markiewski M.M., Lambris J.D. (2007). The role of complement in inflammatory diseases from behind the scenes into the spotlight. Am. J. Pathol..

[71] Ekiri A.B., Landblom D., Doetkott D., Olet S., Shelver W.L., Khaitsa M.L. (2014). Isolation and characterization of Shiga toxin–producing *Escherichia coli* serogroups O26, O45, O103, O111, O113, O121, O145, and O157 shed from range and feedlot cattle from postweaning to slaughter. J. Food Prot..

[72] Ryu J.-H., Beuchat L.R. (2005). Biofilm Formation by *Escherichia coli* O157:H7 on Stainless Steel: Effect of Exopolysaccharide and Curli Production on Its Resistance to Chlorine. Appl. Environ. Microbiol..

[73] Barnhart M.M., Chapman M.R. (2006). Curli biogenesis and function. Annu. Rev. Microbiol..

[74] Vidal O., Longin R., Prigent-Combaret C., Dorel C., Hooreman M., Lejeune P. (1998). Isolation of an *Escherichia coli* K-12 mutant strain able to form biofilms on inert surfaces: Involvement of a new ompR allele that increases curli expression. J. Bacteriol..

[75] Carter M.Q., Feng D., Li H.H. (2019). Curli fimbriae confer shiga toxin-producing *Escherichia coli* a competitive trait in mixed biofilms. Food Microbiol..

[76] Bumunang E.W., Ateba C.N., Stanford K., McAllister T.A., Niu Y.D. (2020). Biofilm formation by South African non-O157 Shiga toxigenic *Escherichia coli* on stainless steel coupons. Can. J. Microbiol..

[77] Jeter C., Matthysse A.G. (2005). Characterization of the binding of diarrheagenic strains of *E. coli* to plant surfaces and the role of curli in the interaction of the bacteria with alfalfa sprouts. Mol. Plant-Microbe Interact..

[78] Hammar M.r., Arnqvist A., Bian Z., Olsén A., Normark S. (1995). Expression of two csg operons is required for production of fibronectin-and congo red-binding curli polymers in *Escherichia coli* K-12. Mol. Microbiol..

[79] Hammar M., Bian Z., Normark S. (1996). Nucleator-dependent intercellular assembly of adhesive curli organelles in *Escherichia coli*. Proc. Natl. Acad. Sci. USA.

[80] Ferrières L., Clarke D.J. (2003). The RcsC sensor kinase is required for normal biofilm formation in *Escherichia coli* K-12 and controls the expression of a regulon in response to growth on a solid surface. Mol. Microbiol..

[81] Jubelin G., Vianney A., Beloin C., Ghigo J.-M., Lazzaroni J.-C., Lejeune P., Dorel C. (2005). CpxR/OmpR interplay regulates curli gene expression in response to osmolarity in *Escherichia coli*. J. Bacteriol..

[82] Martin A., Beutin L. (2011). Characteristics of Shiga toxin-producing *Escherichia coli* from meat and milk products of different origins and association with food producing animals as main contamination sources. Int. J. Food Microbiol..

[83] Watanabe Y., Ozasa K., Mermin J.H., Griffin P.M., Masuda K., Imashuku S., Sawada T. (1999). Factory outbreak of *Escherichia coli* O157: H7 infection in Japan. Emerg. Infect. Dis..

[84] Hilborn E., Mshar P., Fiorentino T., Dembek Z., Barrett T., Howard R., Cartter M. (2000). An outbreak of *Escherichia coli* O157 [ratio] H7 infections and haemolytic uraemic syndrome associated with consumption of unpasteurized apple cider. Epidemiol. Infect..

[85] Fink R.C., Black E.P., Hou Z., Sugawara M., Sadowsky M.J., Diez-Gonzalez F. (2012). Transcriptional Responses of *Escherichia coli* K-12 and O157:H7 Associated with Lettuce Leaves. Appl. Environ. Microbiol..

[86] Silagyi K., Kim S.-H., Lo Y.M., Wei C.-I. (2009). Production of biofilm and quorum sensing by *Escherichia coli* O157: H7 and its transfer from contact surfaces to meat, poultry, ready-to-eat deli, and produce products. Food Microbiol..

[87] Nesse L.L., Sekse C., Berg K., Johannesen K.C.S., Solheim H., Vestby L.K., Urdahl A.M., Griffiths M.W. (2014). Potentially Pathogenic *Escherichia coli* Can Form a Biofilm under Conditions Relevant to the Food Production Chain. Appl. Environ. Microbiol..

[88] Buvens G., Possé B., De Schrijver K., De Zutter L., Lauwers S., Piérard D. (2011). Virulence profiling and quantification of verocytotoxin-producing *Escherichia coli* O145: H28 and O26: H11 isolated during an ice cream–related hemolytic uremic syndrome outbreak. Foodborne Pathog. Dis..

[89] Erickson M.C., Doyle M.P. (2007). Food as a vehicle for transmission of Shiga toxin–producing *Escherichia coli*. J. Food Prot..

[90] Lewis K. (2010). Persister Cells. Annu. Rev. Microbiol..

[91] Ludwig J.B., Shi X., Shridhar P.B., Roberts E.L., DebRoy C., Phebus R.K., Bai J., Nagaraja T.G. (2020). Multiplex PCR Assays for the Detection of One Hundred and Thirty Seven Serogroups of Shiga Toxin-Producing *Escherichia coli* Associated with Cattle. Front. Cell. Infect. Microbiol..

[92] Zhang X., Payne M., Kaur S., Lan R. (2022). Improved Genomic Identification, Clustering, and Serotyping of Shiga Toxin-Producing *Escherichia coli* Using Cluster/Serotype-Specific Gene Markers. Front. Cell. Infect. Microbiol..

[93] GROUP F.W.S.E. (2019). Hazard identification and characterization: Criteria for categorizing Shiga toxin–producing *Escherichia coli* on a risk basis. J. Food Prot..

[94] Lerouge I., Vanderleyden J. (2002). O-antigen structural variation: Mechanisms and possible roles in animal/plant–microbe interactions. FEMS Microbiol. Rev..

[95] Beutin L., Krause G., Zimmermann S., Kaulfuss S., Gleier K. (2004). Characterization of Shiga toxin-producing *Escherichia coli* strains isolated from human patients in Germany over a 3-year period. J. Clin. Microbiol..

[96] DiLuzio W.R., Turner L., Mayer M., Garstecki P., Weibel D.B., Berg H.C., Whitesides G.M. (2005). *Escherichia coli* swim on the right-hand side. Nature.

[97] Erdem A.L., Avelino F., Xicohtencatl-Cortes J., Girón J.A. (2007). Host protein binding and adhesive properties of H6 and H7 flagella of attaching and effacing *Escherichia coli*. J. Bacteriol..

[98] Momtaz H., Farzan R., Rahimi E., Safarpoor Dehkordi F., Souod N. (2012). Molecular characterization of Shiga toxin-producing *Escherichia coli* isolated from ruminant and donkey raw milk samples and traditional dairy products in Iran. Sci. World J..

[99] Perera A., Clarke C.M., Dykes G.A., Fegan N. (2015). Characterization of Shiga toxigenic *Escherichia coli* O157 and Non-O157 isolates from ruminant feces in Malaysia. BioMed Res. Int..

[100] Brusa V., Restovich V., Galli L., Teitelbaum D., Signorini M., Brasesco H., Londero A., García D., Padola N.L., Superno V. (2017). Isolation and characterization of non-O157 Shiga toxin-producing *Escherichia coli* from beef carcasses, cuts and trimmings of abattoirs in Argentina. PLoS ONE.

[101] Essendoubi S., Stashko N., So I., Gensler G., Rolheiser D., Mainali C. (2019). Prevalence of Shiga toxin-producing *Escherichia coli* (STEC) O157: H7, six non-O157 STECs, and Salmonella on beef carcasses in provincially licensed abattoirs in Alberta, Canada. Food Control.

[102] Gould L.H., Walsh K.A., Vieira A.R., Herman K., Williams I.T., Hall A.J., Cole D. (2013). Surveillance for foodborne disease outbreaks—United States, 1998–2008. Morb. Mortal. Wkly. Rep. Surveill. Summ..

[103] Hussein H.S. (2007). Prevalence and pathogenicity of Shiga toxin-producing *Escherichia coli* in beef cattle and their products1,2. J. Anim. Sci..

[104] Gökmen M., İlhan Z., Tavşanlı H., Önen A., Ektik N., Göçmez E.B. (2022). Prevalence and molecular characterization of shiga toxin-producing *Escherichia coli* in animal source foods and green leafy vegetables. Food Sci. Technol. Int..

[105] Bai X., Wang H., Xin Y., Wei R., Tang X., Zhao A., Sun H., Zhang W., Wang Y., Xu Y. (2015). Prevalence and characteristics of Shiga toxin-producing *Escherichia coli* isolated from retail raw meats in China. Int. J. Food Microbiol..

[106] Nong F., Zhang P., Meng J., Xie Q., Li Y., Pan Y., Zhao Y., Liu H. (2021). Characterization of Shiga-toxin producing *Escherichia coli* (STEC) isolated from retail raw meats in Southeast China. Food Control.

[107] (2021). The European Union One Health 2019 Zoonoses Report. Efsa J..

[108] Feng P.C., Delannoy S., Lacher D.W., Bosilevac J.M., Fach P., Beutin L. (2017). Shiga toxin-producing serogroup O91 *Escherichia coli* strains isolated from food and environmental samples. Appl. Environ. Microbiol..

[109] Feng P.C., Delannoy S., Lacher D.W., Dos Santos L.F., Beutin L., Fach P., Rivas M., Hartland E.L., Paton A.W., Guth B.E. (2014). Genetic diversity and virulence potential of Shiga toxin-producing *Escherichia coli* O113: H21 strains isolated from clinical, environmental, and food sources. Appl. Environ. Microbiol..

[110] Valilis E., Ramsey A., Sidiq S., DuPont H.L. (2018). Non-O157 Shiga toxin-producing *Escherichia coli*—A poorly appreciated enteric pathogen: Systematic review. Int. J. Infect. Dis..

[111] Sokolovic M., Šimpraga B., Amšel-Zelenika T., Berendika M., Krstulović F. (2022). Prevalence and Characterization of Shiga Toxin Producing *Escherichia coli* Isolated from Animal Feed in Croatia. Microorganisms.

[112] Yang X., Liu Q., Bai X., Hu B., Jiang D., Jiao H., Lu L., Fan R., Hou P., Matussek A. (2022). High Prevalence and Persistence of *Escherichia coli* Strains Producing Shiga Toxin Subtype 2k in Goat Herds. Microbiol. Spectr..

[113] Yang X., Bai X., Zhang J., Sun H., Fu S., Fan R., He X., Scheutz F., Matussek A., Xiong Y. (2020). *Escherichia coli* strains producing a novel Shiga toxin 2 subtype circulate in China. Int. J. Med. Microbiol..

[114] Pinto G., Sampaio M., Dias O., Almeida C., Azeredo J., Oliveira H. (2021). Insights into the genome architecture and evolution of Shiga toxin encoding bacteriophages of *Escherichia coli*. BMC Genom..

[115] Rodwell E.V., Chan Y.-W., Sawyer C., Carroll A., McNamara E., Allison L., Browning L., Holmes A., Godbole G., McCarthy N. (2022). Shiga toxin-producing *Escherichia coli* clonal complex 32, including serotype O145:H28, in the UK and Ireland. J. Med. Microbiol..

[116] Dewsbury D.M., Renter D.G., Shridhar P.B., Noll L.W., Shi X., Nagaraja T.G., Cernicchiaro N. (2015). Summer and winter prevalence of Shiga toxin–producing *Escherichia coli* (STEC) O26, O45, O103, O111, O121, O145, and O157 in feces of feedlot cattle. Foodborne Pathog. Dis..

[117] Cull C.A., Renter D.G., Dewsbury D.M., Noll L.W., Shridhar P.B., Ives S.E., Nagaraja T.G., Cernicchiaro N. (2017). Feedlot-and pen-level prevalence of enterohemorrhagic *Escherichia coli* in feces of commercial feedlot cattle in two major US cattle feeding areas. Foodborne Pathog. Dis..

[118] Feng P.C., Reddy S. (2013). Prevalences of Shiga toxin subtypes and selected other virulence factors among Shiga-toxigenic *Escherichia coli* strains isolated from fresh produce. Appl. Environ. Microbiol..

[119] Fayemi O.E., Akanni G.B., Elegbeleye J.A., Aboaba O.O., Njage P.M. (2021). Prevalence, characterization and antibiotic resistance of Shiga toxigenic *Escherichia coli* serogroups isolated from fresh beef and locally processed ready-to-eat meat products in Lagos, Nigeria. Int. J. Food Microbiol..

[120] Mora A., López C., Dhabi G., López-Beceiro A.M., Fidalgo L.E., Díaz E.A., Martínez-Carrasco C., Mamani R., Herrera A., Blanco J.E. (2012). Seropathotypes, Phylogroups, Stx Subtypes, and Intimin Types of Wildlife-Carried, Shiga Toxin-Producing *Escherichia coli* Strains with the Same Characteristics as Human-Pathogenic Isolates. Appl. Environ. Microbiol..

[121] Miko A., Pries K., Haby S., Steege K., Albrecht N., Krause G., Beutin L. (2009). Assessment of Shiga toxin-producing *Escherichia coli* isolates from wildlife meat as potential pathogens for humans. Appl. Environ. Microbiol..

[122] Hussein M.A., Merwad A.M., Elabbasy M.T., Suelam I.I., Abdelwahab A.M., Taha M.A. (2019). Prevalence of Enterotoxigenic Staphylococcus aureus and Shiga Toxin Producing *Escherichia coli* in fish in Egypt: Quality parameters and public health hazard. Vector-Borne Zoonotic Dis..

[123] Arthur T.M., Barkocy-Gallagher G.A., Rivera-Betancourt M., Koohmaraie M. (2002). Prevalence and characterization of non-O157 Shiga toxin-producing *Escherichia coli* on carcasses in commercial beef cattle processing plants. Appl. Environ. Microbiol..

[124] Singh P., Sha Q., Lacher D.W., Del Valle J., Mosci R.E., Moore J.A., Scribner K.T., Manning S.D. (2015). Characterization of enteropathogenic and Shiga toxin-producing *Escherichia coli* in cattle and deer in a shared agroecosystem. Front. Cell. Infect. Microbiol..

[125] Asakura H., Makino S.i., Shirahata T., Tsukamoto T., Kurazono H., Ikeda T., Takeshi K. (1998). Detection and genetical characterization of Shiga toxin-producing *Escherichia coli* from wild deer. Microbiol. Immunol..

[126] GM Gonzalez A., MF Cerqueira A. (2020). Shiga toxin-producing *Escherichia coli* in the animal reservoir and food in Brazil. J. Appl. Microbiol..

[127] Díaz-Sánchez S., Sánchez S., Herrera-León S., Porrero C., Blanco J., Dahbi G., Blanco J., Mora A., Mateo R., Hanning I. (2013). Prevalence of Shiga toxin-producing *Escherichia coli*, *Salmonella* spp. and *Campylobacter* spp. in large game animals intended for consumption: Relationship with management practices and livestock influence. Vet. Microbiol..

[128] Fakih I., Thiry D., Duprez J.-N., Saulmont M., Iguchi A., Piérard D., Jouant L., Daube G., Ogura Y., Hayashi T. (2017). Identification of Shiga toxin-producing (STEC) and enteropathogenic (EPEC) *Escherichia coli* in diarrhoeic calves and comparative genomics of O5 bovine and human STEC. Vet. Microbiol..

[129] Beutin L., Martin A. (2012). Outbreak of Shiga toxin–producing *Escherichia coli* (STEC) O104: H4 infection in Germany causes a paradigm shift with regard to human pathogenicity of STEC strains. J. Food Prot..

[130] Shridhar P.B., Siepker C., Noll L.W., Shi X., Nagaraja T., Bai J. (2017). Shiga toxin subtypes of non-O157 *Escherichia coli* serogroups isolated from cattle feces. Front. Cell. Infect. Microbiol..

[131] Capps K.M., Ludwig J.B., Shridhar P.B., Shi X., Roberts E., DebRoy C., Cernicchiaro N., Phebus R.K., Bai J., Nagaraja T. (2021). Identification, Shiga toxin subtypes and prevalence of minor serogroups of Shiga toxin-producing *Escherichia coli* in feedlot cattle feces. Sci. Rep..

[132] Lauzi S., Luzzago C., Chiani P., Michelacci V., Knijn A., Pedrotti L., Corlatti L., Buccheri Pederzoli C., Scavia G., Morabito S. (2022). Free-ranging red deer (*Cervus elaphus*) as carriers of potentially zoonotic Shiga toxin-producing *Escherichia coli*. Transbound. Emerg. Dis..

[133] Milton A.A.P., Agarwal R.K., Priya G.B., Aravind M., Athira C.K., Rose L., Saminathan M., Sharma A.K., Kumar A. (2019). Captive wildlife from India as carriers of Shiga toxin-producing, enteropathogenic and enterotoxigenic *Escherichia coli*. J. Vet. Med. Sci..

[134] Schmidt H., Scheef J.r., Morabito S., Caprioli A., Wieler L.H., Karch H. (2000). A new Shiga toxin 2 variant (Stx2f) from *Escherichia coli* isolated from pigeons. Appl. Environ. Microbiol..

[135] Friesema I.H., Keijzer-Veen M.G., Koppejan M., Schipper H.S., van Griethuysen A.J., Heck M.E., van Pelt W. (2015). Hemolytic uremic syndrome associated with *Escherichia coli* O8: H19 and Shiga toxin 2f gene. Emerg. Infect. Dis..

[136] European Food Safety Authority, European Centre for Disease Prevention and Control (2016). The European Union summary report on trends and sources of zoonoses, zoonotic agents and food-borne outbreaks in 2015. EFSA J..

[137] Dias D., Costa S., Fonseca C., Baraúna R., Caetano T., Mendo S. (2022). Pathogenicity of Shiga toxin-producing *Escherichia coli* (STEC) from wildlife: Should we care?. Sci. Total Environ..

[138] Cooley M.B., Jay-Russell M., Atwill E.R., Carychao D., Nguyen K., Quiñones B., Patel R., Walker S., Swimley M., Pierre-Jerome E. (2013). Development of a robust method for isolation of Shiga toxin-positive *Escherichia coli* (STEC) from fecal, plant, soil and water samples from a leafy greens production region in California. PLoS ONE.

[139] Keen J.E., Wittum T.E., Dunn J.R., Bono J.L., Durso L.M. (2006). Shiga-toxigenic *Escherichia coli* O157 in agricultural fair livestock, United States. Emerg. Infect. Dis..

[140] Duris J.W., Haack S.K., Fogarty L.R. (2009). Gene and Antigen Markers of Shiga-toxin Producing *E. coli* from Michigan and Indiana River Water: Occurrence and Relation to Recreational Water Quality Criteria. J. Environ. Qual..

[141] Prakasan S., Prabhakar P., Lekshmi M., Kumar S., Nayak B.B. (2018). Isolation of Shiga toxin-producing *Escherichia coli* harboring variant Shiga toxin genes from seafood. Vet. World.

[142] Feng P.C., Councell T., Keys C., Monday S.R. (2011). Virulence characterization of Shiga-toxigenic *Escherichia coli* isolates from wholesale produce. Appl. Environ. Microbiol..

[143] Ramaite K., Ekwanzala M.D., Momba M.N.B. (2022). Prevalence and Molecular Characterisation of Extended-Spectrum Beta-Lactamase-Producing Shiga Toxin-Producing *Escherichia coli*, from Cattle Farm to Aquatic Environments. Pathogens.

[144] Elmonir W., Shalaan S., Tahoun A., Mahmoud S.F., Remela E.M.A., Eissa R., El-Sharkawy H., Shukry M., Zahran R.N. (2021). Prevalence, antimicrobial resistance, and genotyping of Shiga toxin-producing *Escherichia coli* in foods of cattle origin, diarrheic cattle, and diarrheic humans in Egypt. Gut Pathog..

[145] Bai X., Hu B., Xu Y., Sun H., Zhao A., Ba P., Fu S., Fan R., Jin Y., Wang H. (2016). Molecular and phylogenetic characterization of non-O157 Shiga toxin-producing *Escherichia coli* strains in China. Front. Cell. Infect. Microbiol..

[146] Fan R., Shao K., Yang X., Bai X., Fu S., Sun H., Xu Y., Wang H., Li Q., Hu B. (2019). High prevalence of non-O157 Shiga toxin-producing *Escherichia coli* in beef cattle detected by combining four selective agars. BMC Microbiol..

[147] Peng Z., Liang W., Hu Z., Li X., Guo R., Hua L., Tang X., Tan C., Chen H., Wang X. (2019). O-serogroups, virulence genes, antimicrobial susceptibility, and MLST genotypes of Shiga toxin-producing *Escherichia coli* from swine and cattle in Central China. BMC Vet. Res..

[148] Fadel H.M., Afifi R., Al-Qabili D.M. (2017). Characterization and zoonotic impact of Shiga toxin producing *Escherichia coli* in some wild bird species. Vet World.

[149] Probert W.S., Miller G.M., Ledin K.E. (2017). Contaminated stream water as source for *Escherichia coli* O157 illness in children. Emerg. Infect. Dis..

[150] Ahlstrom C., Manuel C., Den Bakker H., Wiedmann M., Nightingale K. (2018). Molecular ecology of *Listeria* spp., *Salmonella*, *Escherichia coli* O157: H7 and non-O157 Shiga toxin-producing *E. coli* in pristine natural environments in Northern Colorado. J. Appl. Microbiol..

[151] Dias D., Caetano T., Torres R., Fonseca C., Mendo S. (2019). Shiga toxin-producing *Escherichia coli* in wild ungulates. Sci. Total Environ..

[152] Szczerba-Turek A., Socha P., Bancerz-Kisiel A., Platt-Samoraj A., Lipczynska-Ilczuk K., Siemionek J., Kończyk K., Terech-Majewska E., Szweda W. (2019). Pathogenic potential to humans of Shiga toxin-producing *Escherichia coli* isolated from wild boars in Poland. Int. J. Food Microbiol..

[153] Beutin L., Hammerl J.A., Reetz J., Strauch E. (2013). Shiga toxin-producing *Escherichia coli* strains from cattle as a source of the Stx2a bacteriophages present in enteroaggregative *Escherichia coli* O104:H4 strains. Int. J. Med. Microbiol..

[154] Patel P.N., Lindsey R.L., Garcia-Toledo L., Rowe L.A., Batra D., Whitley S.W., Drapeau D., Stoneburg D., Martin H., Juieng P. (2018). High-Quality Whole-Genome Sequences for 77 Shiga Toxin-Producing *Escherichia coli* Strains Generated with PacBio Sequencing. Genome Announc..

[155] Mellor G.E., Fegan N., Duffy L.L., McMILLAN K.E., Jordan D., Barlow R.S. (2016). National survey of Shiga toxin–producing *Escherichia coli* serotypes O26, O45, O103, O111, O121, O145, and O157 in Australian beef cattle feces. J. Food Prot..

[156] Puri-Giri R., Ghosh A., Thomson J., Zurek L. (2017). House flies in the confined cattle environment carry non-O157 Shiga toxin-producing *Escherichia coli*. J. Med. Entomol..

[157] Mainga A.O., Cenci-Goga B.T., Malahlela M.N., Tshuma T., Kalake A., Karama M. (2018). Occurrence and characterization of seven major Shiga toxin-producing *Escherichia coli* serotypes from healthy cattle on cow–calf operations in South Africa. Zoonoses Public Health.

[158] Dixon A., Cernicchiaro N., Amachawadi R.G., Shi X., Cull C.A., Renter D.G. (2020). Longitudinal characterization of prevalence and concentration of Shiga toxin–producing *Escherichia coli* serogroups in feces of individual feedlot cattle. Foodborne Pathog. Dis..

[159] Engelen F., Thiry D., Devleesschauwer B., Mainil J., De Zutter L., Cox E. (2021). Occurrence of ‘gang of five’ Shiga toxin-producing *Escherichia coli* serogroups on Belgian dairy cattle farms by overshoe sampling. Lett. Appl. Microbiol..

[160] Cernicchiaro N., Oliveira A.R., Hoehn A., Noll L.W., Shridhar P.B., Nagaraja T.G., Ives S.E., Renter D.G., Sanderson M.W. (2020). Associations between season, processing plant, and hide cleanliness scores with prevalence and concentration of major shiga toxin–producing *Escherichia coli* on beef cattle hides. Foodborne Pathog. Dis..

[161] Ballem A., Gonçalves S., Garcia-Meniño I., Flament-Simon S.C., Blanco J.E., Fernandes C., Saavedra M.J., Pinto C., Oliveira H., Blanco J. (2020). Prevalence and serotypes of Shiga toxin-producing *Escherichia coli* (STEC) in dairy cattle from Northern Portugal. PLoS ONE.

[162] Sanches L.A., Gomes M.d.S., Teixeira R.H.F., Cunha M.P.V., Oliveira M.G.X.d., Vieira M.A.M., Gomes T.A.T., Knobl T. (2017). Captive wild birds as reservoirs of enteropathogenic *E. coli* (EPEC) and Shiga-toxin producing *E. coli* (STEC). Braz. J. Microbiol..

[163] Rahman M.M., Lim S.J., Kim W.H., Cho J.Y., Park Y.C. (2020). Prevalence data of diarrheagenic *E. coli* in the fecal pellets of wild rodents using culture methods and PCR assay. Data Brief.

[164] Haindongo N., Nkandi J., Hamatui N., Akai L.A., Hemberger M.Y., Khaiseb S., Molini U. (2018). The prevalence of non-O157: H7 Shiga toxin-producing *Escherichia coli* (STEC) in Namibian game meat. Vet. Ital..

[165] Reimoser F., Putman R. (2011). Impacts of wild ungulates on vegetation: Costs and benefits. Ungulate Management in Europe: Problems and Practices.

[166] Niewiadomska K., Kosicka-Gębska M., Gębski J., Gutkowska K., Jeżewska-Zychowicz M., Sułek M. (2020). Game meat consumption—Conscious choice or just a game?. Foods.

[167] Hedman H.D., Varga C., Duquette J., Novakofski J., Mateus-Pinilla N.E. (2020). Food Safety Considerations Related to the Consumption and Handling of Game Meat in North America. Vet. Sci..

[168] Díaz-Sánchez S., Sánchez S., Sánchez M., Herrera-León S., Hanning I., Vidal D. (2012). Detection and characterization of Shiga toxin-producing *Escherichia coli* in game meat and ready-to-eat meat products. Int. J. Food Microbiol..

[169] Reinstein S., Fox J., Shi X., Alam M., Nagaraja T. (2007). Prevalence of *Escherichia coli* O157: H7 in the American bison (*Bison bison*). J. Food Prot..

[170] Vu-Khac H., Cornick N.A. (2008). Prevalence and genetic profiles of Shiga toxin-producing *Escherichia coli* strains isolated from buffaloes, cattle, and goats in central Vietnam. Vet. Microbiol..

[171] Roug A., Byrne B.A., Conrad P.A., Miller W. (2013). Zoonotic fecal pathogens and antimicrobial resistance in county fair animals. Comp. Immunol. Microbiol. Infect. Dis..

[172] Strachan N.J.C., Rotariu O., Lopes B., MacRae M., Fairley S., Laing C., Gannon V., Allison L.J., Hanson M.F., Dallman T. (2015). Whole Genome Sequencing demonstrates that Geographic Variation of *Escherichia coli* O157 Genotypes Dominates Host Association. Sci. Rep..

[173] Booher S., Cornick N., Moon H. (2002). Persistence of *Escherichia coli* O157: H7 in experimentally infected swine. Vet. Microbiol..

[174] Siddhnath K., Majumdar R., Parhi J., Sharma S., Mehta N., Laishram M. (2018). Detection and characterization of Shiga toxin-producing *Escherichia coli* from carps from integrated aquaculture system. Aquaculture.

[175] Bertelloni F., Lunardo E., Rocchigiani G., Ceccherelli R., Ebani V. (2019). Occurrence of *Escherichia coli* virulence genes in feces of wild birds from Central Italy. Asian Pac. J. Trop. Med..

[176] Smith O.M., Olimpi E.M., Navarro-Gonzalez N., Cornell K.A., Frishkoff L.O., Northfield T.D., Bowles T.M., Edworthy M., Eilers J., Fu Z. (2022). A trait-based framework for predicting foodborne pathogen risk from wild birds. Ecol. Appl..

[177] Kusumoto M., Hikoda Y., Fujii Y., Murata M., Miyoshi H., Ogura Y., Gotoh Y., Iwata T., Hayashi T., Akiba M. (2016). Emergence of a multidrug-resistant Shiga toxin-producing enterotoxigenic *Escherichia coli* lineage in diseased swine in Japan. J. Clin. Microbiol..

[178] Mohamed M.-Y.I., Abu J., Zakaria Z., Khan A.R., Abdul Aziz S., Bitrus A.A., Habib I. (2022). Multi-Drug Resistant Pathogenic *Escherichia coli* Isolated from Wild Birds, Chicken, and the Environment in Malaysia. Antibiotics.

[179] Moore P., Evenson A., Luckey T., McCoy E., Elvehjem C., Hart E. (1946). Use of sulfasuxidine, streptothricin, and streptomycin in nutritional studies with the chick. J. Biol. Chem.

[180] Castanon J. (2007). History of the use of antibiotic as growth promoters in European poultry feeds. Poult. Sci..

[181] Cameron A., McAllister T.A. (2016). Antimicrobial usage and resistance in beef production. J. Anim. Sci. Biotechnol..

[182] Kim J.C., Chui L., Wang Y., Shen J., Jeon B. (2016). Expansion of Shiga Toxin-Producing *Escherichia coli* by Use of Bovine Antibiotic Growth Promoters. Emerg. Infect. Dis..

[183] Iweriebor B.C., Iwu C.J., Obi L.C., Nwodo U.U., Okoh A.I. (2015). Multiple antibiotic resistances among Shiga toxin producing *Escherichia coli* O157 in feces of dairy cattle farms in Eastern Cape of South Africa. BMC Microbiol..

[184] Srinivasan V., Nguyen L.T., Headrick S.I., Murinda S.E., Oliver S.P. (2007). Antimicrobial resistance patterns of Shiga toxin-producing *Escherichia coli* O157: H7 and O157: H7− from different origins. Microb. Drug Resist..

[185] Schroeder C.M., Meng J., Zhao S., DebRoy C., Torcolini J., Zhao C., McDermott P.F., Wagner D.D., Walker R.D., White D.G. (2002). Antimicrobial resistance of *Escherichia coli* O26, O103, O111, O128, and O145 from animals and humans. Emerg. Infect. Dis..

[186] Tamang M.D., Sunwoo H., Jeon B. (2017). Phage-mediated dissemination of virulence factors in pathogenic bacteria facilitated by antibiotic growth promoters in animals: A perspective. Anim. Health Res. Rev..

[187] McGannon C.M., Fuller C.A., Weiss A.A. (2010). Different classes of antibiotics differentially influence Shiga toxin production. Antimicrob. Agents Chemother..

[188] Liu Y.-Y., Wang Y., Walsh T.R., Yi L.-X., Zhang R., Spencer J., Doi Y., Tian G., Dong B., Huang X. (2016). Emergence of plasmid-mediated colistin resistance mechanism MCR-1 in animals and human beings in China: A microbiological and molecular biological study. Lancet Infect. Dis..

[189] Shafiq M., Rahman S.U., Bilal H., Ullah A., Noman S.M., Zeng M., Yuan Y., Xie Q., Li X., Jiao X. (2022). Incidence and molecular characterization of ESBL-producing and colistin-resistant *Escherichia coli* isolates recovered from healthy food-producing animals in Pakistan. J. Appl. Microbiol..

[190] Shafiq M., Huang J., Shah J.M., Ali I., Rahman S.U., Wang L. (2021). Characterization and resistant determinants linked to mobile elements of ESBL-producing and mcr-1-positive *Escherichia coli* recovered from the chicken origin. Microb. Pathog..

[191] Rahal E.A., Fadlallah S.M., Nassar F.J., Kazzi N., Matar G.M. (2015). Approaches to treatment of emerging Shiga toxin-producing *Escherichia coli* infections highlighting the O104: H4 serotype. Front. Cell. Infect. Microbiol..

[192] Ochoa T.J., Chen J., Walker C.M., Gonzales E., Cleary T.G. (2007). Rifaximin does not induce toxin production or phage-mediated lysis of Shiga toxin-producing *Escherichia coli*. Antimicrob. Agents Chemother..

[193] Kurioka T., Yunou Y., Harada H., Kita E. (1999). Efficacy of antibiotic therapy for infection with Shiga-like toxin-producing *Escherichia coli* O157: H7 in mice with protein-calorie malnutrition. Eur. J. Clin. Microbiol. Infect. Dis..

[194] Mir R.A., Kudva I.T. (2019). Antibiotic-resistant Shiga toxin-producing *Escherichia coli*: An overview of prevalence and intervention strategies. Zoonoses Public Health.

[195] Jost C., Bidet P., Carrere T., Mariani-Kurkdjian P., Bonacorsi S. (2016). Susceptibility of enterohaemorrhagic *Escherichia coli* to azithromycin in France and analysis of resistance mechanisms. J. Antimicrob. Chemother..

[196] Wang Y., Subedi D., Li J., Wu J., Ren J., Xue F., Dai J., Barr J.J., Tang F. (2022). Phage Cocktail Targeting STEC O157: H7 Has Comparable Efficacy and Superior Recovery Compared with Enrofloxacin in an Enteric Murine Model. Microbiol. Spectr..

[197] Wu J., Zeng H., Qian X., Li Y., Xue F., Ren J., Dai J., Tang F. (2022). Pre-treatment with phages achieved greater protection of mice against infection with Shiga toxin-producing *Escherichia coli* than post-treatment. Res. Vet. Sci..

[198] Luna S., Krishnasamy V., Saw L., Smith L., Wagner J., Weigand J., Tewell M., Kellis M., Penev R., McCullough L. (2018). Outbreak of *E. coli* O157: H7 infections associated with exposure to animal manure in a rural community—Arizona and Utah, June–July 2017. Morb. Mortal. Wkly. Rep..

[199] Venegas-Vargas C., Henderson S., Khare A., Mosci R.E., Lehnert J.D., Singh P., Ouellette L.M., Norby B., Funk J.A., Rust S. (2016). Factors associated with Shiga toxin-producing *Escherichia coli* shedding by dairy and beef cattle. Appl. Environ. Microbiol..

[200] Mechie S., Chapman P., Siddons C. (1997). A fifteen month study of *Escherichia coli* O157: H7 in a dairy herd. Epidemiol. Infect..

[201] Braden K., Blanton Jr J., Allen V., Pond K., Miller M. (2004). *Ascophyllum nodosum* supplementation: A preharvest intervention for reducing *Escherichia coli* O157: H7 and Salmonella spp. in feedlot steers. J. Food Prot..

[202] Walle K.V., Vanrompay D., Cox E. (2013). Bovine innate and adaptive immune responses against *Escherichia coli* O157: H7 and vaccination strategies to reduce faecal shedding in ruminants. Vet. Immunol. Immunopathol..

[203] Snedeker K.G., Campbell M., Sargeant J.M. (2012). A systematic review of vaccinations to reduce the shedding of *Escherichia coli* O157 in the faeces of domestic ruminants. Zoonoses Public Health.

[204] Blazar J., Allard M., Lienau E.K. (2011). Insects as vectors of foodborne pathogenic bacteria. Terr. Arthropod Rev..

[205] Ray R., Potts R., Pietri J.E. (2020). The persistence of *Escherichia coli* infection in German cockroaches (Blattodea: Blattellidae) varies between host developmental stages and is influenced by the gut microbiota. J. Med. Entomol..

[206] Sánchez S., Martínez R., García A., Vidal D., Blanco J., Blanco M., Blanco J., Mora A., Herrera-León S., Echeita A. (2010). Detection and characterisation of O157: H7 and non-O157 Shiga toxin-producing *Escherichia coli* in wild boars. Vet. Microbiol..

[207] Bakry N., Awad W., Ahmed S., Kamel M. (2022). The role of Musca domestica and milk in transmitting pathogenic multidrug-resistant *Escherichia coli* and associated phylogroups to neonatal calves. Environ. Sci. Pollut. Res..

[208] Perrat A., Branchu P., Decors A., Turci S., Bayon-Auboyer M.-H., Petit G., Grosbois V., Brugère H., Auvray F., Oswald E. (2022). Wild Boars as Reservoir of Highly Virulent Clone of Hybrid Shiga Toxigenic and Enterotoxigenic *Escherichia coli* Responsible for Edema Disease, France. Emerg. Infect. Dis..

[209] Zhu J.J., Li A.Y., Pritchard S., Tangtrakulwanich K., Baxendale F.P., Brewer G. (2011). Contact and fumigant toxicity of a botanical-based feeding deterrent of the stable fly, Stomoxys calcitrans (Diptera: Muscidae). J. Agric. Food Chem..

[210] Pavela R. (2007). Lethal and sublethal effects of thyme oil (Thymus vulgaris L.) on the house fly (Musca domestica Lin.). J. Essent. Oil Bear. Plants.

[211] Weerarathne P., Payne J., Saha J., Kountoupis T., Jadeja R., Jaroni D. (2021). Evaluating the efficacy of sodium acid sulfate to reduce *Escherichia coli* O157: H7 and its biofilms on food-contact surfaces. LWT.

[212] Sloup R.E., Cieza R.J., Needle D.B., Abramovitch R.B., Torres A.G., Waters C.M. (2016). Polysorbates prevent biofilm formation and pathogenesis of *Escherichia coli* O104: H4. Biofouling.

[213] Barbieri R., Coppo E., Marchese A., Daglia M., Sobarzo-Sánchez E., Nabavi S.F., Nabavi S.M. (2017). Phytochemicals for human disease: An update on plant-derived compounds antibacterial activity. Microbiol. Res..

[214] Lillehoj H., Liu Y., Calsamiglia S., Fernandez-Miyakawa M.E., Chi F., Cravens R.L., Oh S., Gay C.G. (2018). Phytochemicals as antibiotic alternatives to promote growth and enhance host health. Vet. Res..

[215] Liu H., Liu Y., Hu L., Suo Y., Zhang L., Jin F., Feng X., Teng N., Li Y. (2014). Effects of dietary supplementation of quercetin on performance, egg quality, cecal microflora populations, and antioxidant status in laying hens. Poult. Sci..

[216] Lee J.-H., Regmi S.C., Kim J.-A., Cho M.H., Yun H., Lee C.-S., Lee J. (2011). Apple flavonoid phloretin inhibits *Escherichia coli* O157: H7 biofilm formation and ameliorates colon inflammation in rats. Infect. Immun..

[217] Lee J.-H., Cho H.S., Joo S.W., Chandra Regmi S., Kim J.-A., Ryu C.-M., Ryu S.Y., Cho M.H., Lee J. (2013). Diverse plant extracts and trans-resveratrol inhibit biofilm formation and swarming of *Escherichia coli* O157: H7. Biofouling.

[218] Lee J.-H., Kim Y.-G., Ryu S.Y., Cho M.H., Lee J. (2014). Ginkgolic acids and Ginkgo biloba extract inhibit *Escherichia coli* O157: H7 and Staphylococcus aureus biofilm formation. Int. J. Food Microbiol..

[219] Lee J.-H., Kim Y.-G., Cho H.S., Ryu S.Y., Cho M.H., Lee J. (2014). Coumarins reduce biofilm formation and the virulence of *Escherichia coli* O157: H7. Phytomedicine.

[220] Kim Y.-G., Lee J.-H., Gwon G., Kim S.-I., Park J.G., Lee J. (2016). Essential oils and eugenols inhibit biofilm formation and the virulence of *Escherichia coli* O157: H7. Sci. Rep..

[221] Moretti M.D., Sanna-Passino G., Demontis S., Bazzoni E. (2002). Essential oil formulations useful as a new tool for insect pest control. AAPs PharmSciTech.

[222] Kholif A.E., Olafadehan O.A. (2021). Essential oils and phytogenic feed additives in ruminant diet: Chemistry, ruminal microbiota and fermentation, feed utilization and productive performance. Phytochem. Rev..

[223] Xu Y., Dugat-Bony E., Zaheer R., Selinger L., Barbieri R., Munns K., McAllister T.A., Selinger L.B. (2014). *Escherichia coli* O157:H7 Super-Shedder and Non-Shedder Feedlot Steers Harbour Distinct Fecal Bacterial Communities. PLoS ONE.

[224] Zaheer R., Dugat-Bony E., Holman D., Cousteix E., Xu Y., Munns K., Selinger L.J., Barbieri R., Alexander T., McAllister T.A. (2017). Changes in bacterial community composition of *Escherichia coli* O157: H7 super-shedder cattle occur in the lower intestine. PLoS ONE.

[225] Mir R.A., Schaut R.G., Looft T., Allen H.K., Sharma V.K., Kudva I.T. (2020). Recto-anal junction (RAJ) and fecal microbiomes of cattle experimentally challenged with *Escherichia coli* O157: H7. Front. Microbiol..

[226] Wang O., McAllister T.A., Plastow G., Stanford K., Selinger B., Guan L.L. (2018). Interactions of the Hindgut Mucosa-Associated Microbiome with Its Host Regulate Shedding of *Escherichia coli* O157:H7 by Cattle. Appl. Environ. Microbiol..

[227] Chen X., Yan F., Liu T., Zhang Y., Li X., Wang M., Zhang C., Xu X., Deng L., Yao J. (2022). Ruminal Microbiota Determines the High-Fiber Utilization of Ruminants: Evidence from the Ruminal Microbiota Transplant. Microbiol. Spectr..

[228] Sha Y., Hu J., Shi B., Dingkao R., Wang J., Li S., Zhang W., Luo Y., Liu X. (2020). Characteristics and functions of the rumen microbial community of Cattle-Yak at different ages. BioMed Res. Int..

[229] Saleem F., Ametaj B.N., Bouatra S., Mandal R., Zebeli Q., Dunn S.M., Wishart D.S. (2012). A metabolomics approach to uncover the effects of grain diets on rumen health in dairy cows. J. Dairy Sci..

[230] Liu J., Liu F., Cai W., Jia C., Bai Y., He Y., Zhu W., Li R.W., Song J. (2020). Diet-induced changes in bacterial communities in the jejunum and their associations with bile acids in Angus beef cattle. Anim. Microbiome.

[231] Keen J., Uhlich G., Elder R. Effects of hay-and grain-based diets on fecal shedding in naturally-acquired enterohemorrhagic *E. coli* (EHEC) O157 in beef feedlot cattle. Proceedings of the 80th Conference Research Workers in Animal Diseases.

[232] Jacob M., Paddock Z., Renter D.G., Lechtenberg K., Nagaraja T. (2010). Inclusion of dried or wet distillers’ grains at different levels in diets of feedlot cattle affects fecal shedding of *Escherichia coli* O157: H7. Appl. Environ. Microbiol..

[233] Gilbert J.A., Blaser M.J., Caporaso J.G., Jansson J.K., Lynch S.V., Knight R. (2018). Current understanding of the human microbiome. Nat. Med..

[234] Rinninella E., Raoul P., Cintoni M., Franceschi F., Miggiano G.A.D., Gasbarrini A., Mele M.C. (2019). What is the healthy gut microbiota composition? A changing ecosystem across age, environment, diet, and diseases. Microorganisms.

[235] Kim A. (2015). Dysbiosis: A review highlighting obesity and inflammatory bowel disease. J. Clin. Gastroenterol..

[236] Singh P., Teal T.K., Marsh T.L., Tiedje J.M., Mosci R., Jernigan K., Zell A., Newton D.W., Salimnia H., Lephart P. (2015). Intestinal microbial communities associated with acute enteric infections and disease recovery. Microbiome.

[237] Mendes R., Raaijmakers J.M. (2015). Cross-kingdom similarities in microbiome functions. ISME J..

[238] Yu L.C.-H., Shih Y.-A., Wu L.-L., Lin Y.-D., Kuo W.-T., Peng W.-H., Lu K.-S., Wei S.-C., Turner J.R., Ni Y.-H. (2014). Enteric dysbiosis promotes antibiotic-resistant bacterial infection: Systemic dissemination of resistant and commensal bacteria through epithelial transcytosis. Am. J. Physiol.-Gastrointest. Liver Physiol..

[239] Winter S.E., Winter M.G., Xavier M.N., Thiennimitr P., Poon V., Keestra A.M., Laughlin R.C., Gomez G., Wu J., Lawhon S.D. (2013). Host-derived nitrate boosts growth of *E. coli* in the inflamed gut. Science.

[240] Tarr G., Shringi S., Oltean H., Mayer J., Rabinowitz P., Wakefield J., Tarr P., Besser T., Phipps A. (2018). Importance of case age in the purported association between phylogenetics and hemolytic uremic syndrome in *Escherichia coli* O157: H7 infections. Epidemiol. Infect..

[241] Gigliucci F., von Meijenfeldt F.B., Knijn A., Michelacci V., Scavia G., Minelli F., Dutilh B.E., Ahmad H.M., Raangs G.C., Friedrich A.W. (2018). Metagenomic characterization of the human intestinal microbiota in fecal samples from STEC-infected patients. Front. Cell. Infect. Microbiol..

[242] Lee K.-S., Jeong Y.-J., Lee M.-S. (2021). *Escherichia coli* Shiga toxins and gut microbiota interactions. Toxins.

[243] Saito K., Suzuki R., Koyanagi Y., Isogai H., Yoneyama H., Isogai E. (2019). Inhibition of enterohemorrhagic *Escherichia coli* O157: H7 infection in a gnotobiotic mouse model with pre-colonization by Bacteroides strains. Biomed. Rep..

[244] Zumbrun S.D., Melton-Celsa A.R., Smith M.A., Gilbreath J.J., Merrell D.S., O’Brien A.D. (2013). Dietary choice affects Shiga toxin-producing *Escherichia coli* (STEC) O157:H7 colonization and disease. Proc. Natl. Acad. Sci. USA.

[245] Byres E., Paton A.W., Paton J.C., Löfling J.C., Smith D.F., Wilce M.C., Talbot U.M., Chong D.C., Yu H., Huang S. (2008). Incorporation of a non-human glycan mediates human susceptibility to a bacterial toxin. Nature.

[246] Paton A.W., Paton J.C. (2010). *Escherichia coli* Subtilase Cytotoxin. Toxins.

[247] Varki N.M., Varki A. (2007). Diversity in cell surface sialic acid presentations: Implications for biology and disease. Lab. Investig..

[248] Kim Y., Hwang S.W., Kim S., Lee Y.-S., Kim T.-Y., Lee S.-H., Kim S.J., Yoo H.J., Kim E.N., Kweon M.-N. (2020). Dietary cellulose prevents gut inflammation by modulating lipid metabolism and gut microbiota. Gut Microbes.

[249] Beddoe T., Paton A.W., Le Nours J., Rossjohn J., Paton J.C. (2010). Structure, biological functions and applications of the AB5 toxins. Trends Biochem. Sci..

[250] Su Z., Zhang L., Sun H., Hu Y., Fanning S., Du P., Cui S., Bai L. (2021). Characterization of non-O157 Shiga toxin-producing *Escherichia coli* cultured from cattle farms in Xinjiang Uygur Autonomous Region, China, during 2016–2017. Foodborne Pathog. Dis..

[251] Brooks J.T., Sowers E.G., Wells J.G., Greene K.D., Griffin P.M., Hoekstra R.M., Strockbine N.A. (2005). Non-O157 Shiga toxin–producing *Escherichia coli* infections in the United States, 1983–2002. J. Infect. Dis..

[252] Khan A., Das S., Ramamurthy T., Sikdar A., Khanam J., Yamasaki S., Takeda Y., Nair G.B. (2002). Antibiotic resistance, virulence gene, and molecular profiles of Shiga toxin-producing *Escherichia coli* isolates from diverse sources in Calcutta, India. J. Clin. Microbiol..

[253] Mora A., Herrrera A., López C., Dahbi G., Mamani R., Pita J.M., Alonso M.P., Llovo J., Bernárdez M.I., Blanco J.E. (2011). Characteristics of the Shiga-toxin-producing enteroaggregative *Escherichia coli* O104: H4 German outbreak strain and of STEC strains isolated in Spain. Int Microbiol.

[254] Withenshaw S.M., Smith R.P., Davies R., Smith A.E., Gray E., Rodgers J. (2022). A systematized review and qualitative synthesis of potential risk factors associated with the occurrence of non-O157 Shiga toxin-producing *Escherichia coli* (STEC) in the primary production of cattle. Compr. Rev. Food Sci. Food Saf..

[255] Menrath A., Wieler L.H., Heidemanns K., Semmler T., Fruth A., Kemper N. (2010). Shiga toxin producing *Escherichia coli*: Identification of non-O157: H7-Super-Shedding cows and related risk factors. Gut Pathog..

[256] Allué-Guardia A., Martínez-Castillo A., Muniesa M. (2014). Persistence of infectious Shiga toxin-encoding bacteriophages after disinfection treatments. Appl. Environ. Microbiol..

[257] Solheim H., Sekse C., Urdahl A.M., Wasteson Y., Nesse L.L. (2013). Biofilm as an environment for dissemination of stx genes by transduction. Appl. Environ. Microbiol..

